# Vessel sign analysis paves the way to optimized CBCT application in interventional pulmonology: COMBINED algorithm as a one-stop-shop

**DOI:** 10.7150/jca.109996

**Published:** 2025-03-21

**Authors:** Wolfgang Hohenforst-Schmidt, Ying Xu, Julia Greeven, Sander Langereis, Haidong Huang, Jian Liu, Xiaopeng Yao, Xiaping Shen, Yang Yang, Liangquan Wu, Paul Zarogoulidis, Stamatis Petousis, Chrysoula Margioula-Siarkou, Dimitris Petridis, Michael Steinheimer, Andreas Riedel, Noufal Aboobaker, Evaggelos Karamitrousis, Eleni-Isidora Perdikouri, Anastasios Vagionas, Thomas Vogl, Anil Sinha

**Affiliations:** 1 Thorax Centre Südwestfalen, Märkische Kliniken, ''Lüdenscheid'' Clinics, aff. University of Bonn and Private University of Hamburg, Germany.; 2 Sana Clinic Group Franken, Department of Cardiology / Pulmonology / Intensive Care / Nephrology, 'Hof'' Clinics, University of Erlangen, Hof, Germany.; 3 Department of Respiratory and Critical Care Medicine, Shanghai Ninth People's Hospital, Shanghai Jiao Tong University School of Medicine, Shanghai, China.; 4 Technical Medicine, Leiden University Medical Center; Delft University of Technology; Erasmus University Medical Center Rotterdam, The Netherlands.; 5 Department of Clinical Science IGT-S, Philips Medical System, Best, The Netherlands.; 6 Department of Respiratory and Critical Care Medicine, The First Affiliated Hospital of Naval Medical University (Shanghai Changhai hospital), Shanghai, China.; 7 Department of Radiology, The First Affiliated Hospital of Naval Medical University (Shanghai Changhai hospital), Shanghai, China.; 8 Department of Respiratory and Critical Care Medicine, The Affiliated Jiangning Hospital of Nanjing Medical University, Nanjing 211100, China.; 9 Pulmonary Department-Oncology Unit, General Clinic Euromedica, Thessaloniki, Greece.; 10 Obstetric department, Hippokrateio University Hospital, Aristotle University of Thessaloniki, Thessaloniki, Greece.; 11 Department of Food Technology, Hellenic International University, Thessaloniki, Greece.; 12 Onocology Department, G. Papageorgiou Univerity Hospital, Aristotle University of Thessaloniki, Thessaloniki, Greece.; 13 Oncology Department, General Hospital of Volos, Volos, Greece.; 14 Department of Diagnostic and Interventional Radiology, Goethe University of Frankfurt, Frankfurt, Germany.; 15 Oncology Department, General Hospital of Kavala, Kavala, Greece.

**Keywords:** cone beam CT, computed tomography, radial-ebus, transbronchial biopsy, cryobiopsy, vessel sign

## Abstract

**Introduction:** We used CBCT application as one-stop-shop nodule orientated approach in regards to increase DY, reduce complication rate, reduce time on-table and economical costs with classical peripheral instruments including mini-cryoprobe (ERBE 1,1mm), rEBUS (Olympus) and standard RUFBs (Olympus Company) with at least 2mm working channel and 4,2mm outer diameter for the diagnosis of peripheral targets (iSPNs) in a prospective all-comers registry after detailed analysis of pre-interventional CT for vessel- and bronchus sign classes.

**Materials and Methods:** From Jun 2017 until Nov 2019 in 90 all-comers patients between 16 and 95 years fit for bronchoscopy with 101 peripheral lesions in a daily routine scheme after informed consent about this prospective registry were included. For histological proven benign disease in any lesion patients had to adhere FU according radiological guidelines and further on by re-visits for at least 2 years after biopsy resulting into last visit in Feb 2022 without any drop-out. Present HRCT was mandatory to achieve one day before intervention. It had to be decided by the examiner mainly after analysis of the preset HRCT which of the 3 CBCT driven modalities were used for diagnostical approach: A) Pure endobronchial approach (CBCT, rEBUS, TBB), B) Pure transthoracical approach with a 21G core-biopsy needle (BIOPINCE needle) with CBCT only, or C) Combined approach as described below (CBCT, rEBUS, TTNA). As instruments were available common forceps and needles, EWC, curette and various RUFB (Olympus Company) mentioned in the materials section. A second CBCT was only allowed in the combined approach group to plan the 3D transthoracic approach in expiration whereas even a CBCT for tool-in-lesion control (TIL CBCT) was never allowed in all 3 groups.

**Results:** In 100 lesions predefined modalities pure endobiopsy, pure TTNA and combined approaches were performed in 77, 9 and 14 lesions respectively without any pneumothorax or bleeding. In these 3 modalities we found confirmed (mostly specific) benign and malignant cases 47 and 30, 4 and 5, 2 and 12 respectively. Lesion sizes in the 3 different groups were (median, mean) 14 and 17,7mm (of those 41 invisible of 77 under XR (53%) in the pure endobiopsy group), 27 and 31mm (11% invisible under XR in the pure TTNA group), 18,5 and 23mm (35% invisible under XR in the combined group) respectively. In the 3 groups for the malignant cases 25 of 30, 5 of 5 and 12 of 12 were diagnosed correctly rendering a diagnostical yield of 42 in 47 malignant cases for the whole algorithm (89,4%) with sizes (mean, median) for the whole algorithm of 16 and 19,7mm respectively which is comparable to published data for robotic-assisted bronchoscopy yield. In regards to vessel sign analysis it has to be clearly stated that the significance level for outcome prediction is inferior to bronchus sign analysis. In multivariate analysis there was a clear tendency towards higher outcome prediction especially if a pulmonary artery branch leads into such target even when a bronchus sign is missing. For NY when comparing univariate analysis and partition model analysis at a set diameter of >11mm with significance (p=0,0052) the additional advantage of analysing a given vessel sign (especially pulmonary artery branches) seems to add on 19% of valuable outcome prediction.

**Conclusion:** A nodule orientated approach in a manual CBCT-AF environment including typical instruments renders in experienced hands comparable results to robotic assisted bronchoscopy even without UTN bronchoscopes or other specialized, therefore expensive tools. In multivariate analysis only bronchus sign analysis revealed significant (p = 0,05) prediction of navigational yield outcome prediction whereas vessel sign analysis increases highly the odds ratio in favor of positive outcome prediction but without significance at the given level. In a partition model to erase outliers at a set iSPN diameter >11mm vessel sign analysis (especially pulmonary artery branches) renders a significant and ameliorated prediction of NY.

## Introduction

The pulmonary nodule is the most difficult radiologic finding that a medical physician has to investigate^1^. Until recently due to lack of diagnostic instruments we used to perform biopsy under computed tomography guidance resulting in many cases in negative results or complications such as pneumothorax^2^. In the last 15 years we have the ability to perform endoscopically biopsies to pulmonary nodules with the use of radial-endobronchial ultrasound (r-EBUS) with or without the combination of radiology equipment^3^,^4^. In the first years we used C-ARM, but later novel radiology equipment were added such as Cone Beam Computer tomography (CBCT)^5^. Moreover; novel navigational equipment were created such as electromagnetic navigation systems^6^. Furthermore; additionally pulmonary physicians were educated to rapid on site evaluation (ROSE) technique, as a result true positive results were raised up to 85% in centers of excellence^7^. The mode of ventilation plays also a crucial rule in best management of patients with chronic obstructive pulmonary disease (COPD) and also in lowering the time of examination^8^. In our study we focused in the investigation of combining different technologies such as radial-EBUS and CBCT as an efficient methodology of pulmonary nodules.

Imaging technology like Cone beam Computer tomography (CBCT) using augmented fluoroscopy (AF) has tremendously improved the on-table visualisation options in Interventional Pulmonology (IP) for increased diagnostical yield (DY) in comparison to the pre-CBCT area 2 decades ago especially in the field of incidental solitary nodules (iSPNs) below 2cm. Today we are talking about an expectable DY of 78% for iSPNs between 10-20mm without repeated CBCT for tool-in-lesion (CBCT TIL) control, robotic use or ROSE^9^. Even in 2014 in the first-in-men published paper about CBCT-AF navigated endobronchial biopsy (including rEBUS) by W. Hohenforst-Schmidt *et al.*^5^ the under fluoroscopy (XR) invisible malignant iSPN subgroup (n=12) of 15mm+3mm diameter (+/-SD) rendered a DY of 75% for malignant cases using only forceps and no specialized tool like cryoprobe, UTN-bronchoscopes, EWC, cross-country device or CBCT TIL which increases up to 12% the expectable DY in comparison w/o CBCT TIL^9^. Furthermore CBCT seems to be key when it comes to potentially curative endobronchial ablation applications with thermal devices or newer techniques like electroporation for navigation, positioning and confirmation of tool-in-lesion. Our group^10^ was the first one publishing on endobronchial application of CBCT-AF in Interventional Pulmonology and the dependency on optimized ventilation strategy which is in our setting still achieved by nasal jet-catheter ventilation so far in the majority of all cases without the help of anaesthesiologist^8^. The knowledge about hyperinflation facilitated CT-imaging was later reported in one study by Ferrer *et al.*^11^ The same aspect is true for CBCT with the difference that one can titrate the working pressure higher along with the diaphragma position during a CBCT-AF navigated bronchoscopy in deeply sedated or generally anaesthesized patients than patients being awake in regular CT imaging. Due to therefore achieved higher working pressure we expect a better contrast with a higher increased number of small peripheral airways than reported by Ferrer although no comparison or literature exists.

## Materials and Methods

### Concept

Our hypothesis was to set up such algorithm in order to take the best out of both worlds - Interventional Pulmonology and Interventional Radiology - with optimisation for time on-table, lowest adverse event rate, lowest financial costs and highest DY - consisting of pure endobronchial approach, pure transthoracic approach and combined approach. This algorithm was guided by analysis of vessel sign (especially pulmonary arterial vessel sign) and bronchus sign (extended Tokoro classification), size and position in regards to critical structure and zone including rEBUS and AF analysis. In all cases it was the decision of the examiner to choose on one of three paths mainly after the analysis of the pre-interventional naive high-resolution CT:

a) Pure endobronchial biopsy was preferred in all cases when an iSPN showed any vessel sign but especially a PA positive VS (2aVS) leading into the center or surrounding of the iSPN and/or a positive bronchus sign aiming at or leading into the center of a lesion. As explained above we rated the presence of a favorable PA positive VS (2aVS) higher than any positive bronchus sign leading into the targeted lesion (2BS) to go for this approach. Transbronchial access technique was allowed only in zone 3 (and not in zone 2 due to bleeding risk) with EWC, curette or any other classical instrument (including ERBE 1,1mm cryoprobe) except for brush. A pure FNA was allowed in zone 2 and 3 for negative BS cases or negative rEBUS-cases.

b) Especially in all negative (missing) BS (0BS) cases due to anatomy a PV positive VS (1VS) is an unfavorable vessel sign to reach an iSPN in a relatively predictable manner with less time consumption on table, the same is expected to be true for a missing vessel sign (0VS) and less true for an undecisive vessel sign (2bVS). In those cases with a (relatively favorable) bronchus sign in the surrounding of the target the decision to move a case in the pure endobronchial biopsy group was achieved by rEBUS- and AF-analysis. If rEBUS even with the help of an EWC did not render at least an inside position we did not take a biopsy but left the bronchoscope in the segmental position (to reduce risk of pneumothorax by blocking airflow) and turned over to transthoracic approach which was only allowed in zone 3. The sequence of both approaches is defined as combined approach (but only 1 biopsy) which is different to the unpublished definition in 2015. Transracial access with a cross-country device or any other aggressive instrumentation was not allowed in a (preplanned) combined approach.

c) In those cases where we did see nor a bronchus neither any vessel sign or only a pulmonary vein branch leading towards the iSPN we tended clearly towards a pure transthoracic approach especially in those cases clearly touching the inner thoracic wall.

From Jun 2017 until Nov 2019 in 90 all-comers patients between 18 and 90 years fit for bronchoscopy with 101 peripheral lesions in a daily routine scheme after informed consent about this prospective registry were included, one lesion had to be excluded due to patient's hemodynamic instability during intervention and were not taken into consideration for statistics (no biopsy, but on FU benign). For benign disease in any lesion patients had to adhere FU according radiological guidelines and further on by re-visits for at least 2 years after biopsy resulting into last visit in Feb 2022 without any drop-out. Preset HRCT was mandatory to achieve one day before intervention. It had to be decided by the examiner mainly after analysis of the preset HRCT which of the 3 CBCT driven modalities were used for diagnostical approach: Pure endobronchial approach (CBCT, rEBUS, TBB or FNA), pure transthoracic approach (CBCT, TTNA) with a 21G core-biopsy needle (BIOPINCE needle) with CBCT only or combined approach as described above (CBCT, rEBUS, TTNA). As instruments were available common forceps and needles, EWC, curette and various RUFB (Olympus Company) mentioned in the materials section. A second CBCT was only allowed in the combined approach group to plan the 3D transthoracic approach whereas even a CBCT for tool-in-lesion control (TIL CBCT) was never allowed in all 3 groups.

In detail we measured (11 criteria) and partially calculated (4 criteria) the following raw data for 100 analyzed lesions:

- Visibility on XR (0,1): A 'case blinded' experienced radiographer had to show the target on first XR on table before any CBCT. There was already a certain bias pro visibility as for each target the detector was at least positioned towards the relevant lung area.

- Bronchus sign (BS) in extended Tokoro-classification on preset naive HRCT or CBCT

• Class 0 = no BS

• Class 1 = BS adjacent to iSPN

• Class 2b = BS leading inside into the outer 1/3 of an iSPN volume

• Class 2a = BS leading centred into the inner 2/3 of an iSPN volume

- rEBUS position for NY

• Class 0 = not touching the iSPN

• Class 1 = adjacent / tangential to the iSPN

• Class 2b = inside the outer 1/3 of the iSPN (which means at least an excentric position)

• Class 2a = centred in the inner 2/3 of the iSPN (which means always at least a concentric position)

• AF was used as additional modality to optimize NY.

- Vessel sign with the following classes

• Class 0 = no vessel touching the iSPN

• Class 1 = a branch of a pulmonary vein touching the iSPN

• Class 2b = a branch of an undecisive vessel touching the iSPN

• Class 2a = a branch of a pulmonary artery touching the iSPN

- DY (TP and FN)

- NY (with 0 not the iSPN reached status and 1 for all other rEBUS-classes except for rEBUS-class 0)

- Malignant / benign iSPN

- lung zone (1-3)

- size in all 3 dimensions, thereby

- calculated average and median of 3 dimensions

- use of TBAT (transbronchial access technique) (0,1): A cross-country device or a burning cath or a needle-balloon technique was not allowed. TBAT was only allowed in lung zone 3 / distal border of lung zone 2 for maximal 3 cm distance.

- calculated 3D-transformation volume (3Dsize, 3D-sphere) by measured size in all 3 dimensions.

- MACE: Any complication increasing hospital stay.

Time on bronchoscopy was not allowed to be longer than 1 hour and on average 47 minutes.

## Materials

## Results

### A) Statistics for endobiopsy group

#### Method data analysis

For each outcome, associations with the corresponding set of variables were checked by Fisher's exact test (for categorical variables), or checked by Wilcoxon-Mann-Whitney test (for continuous variables).

P values <0.05 were considered significant; all tests were two-sided. All statistical analyses were performed in RStudio Team (2020). RStudio: Integrated Development for R. RStudio, PBC, Boston, MA. Version 2022.12.0+353.

Independent correlation of the presence of a bronchus sign and presence of a vessel sign or only PA vessel sign with DY using a multivariate regression analysis was assessed. The interaction between vessel sign and bronchus sign was evaluated using a multinominal regression analysis.

##### I) Univariate analysis (Table 3, 4, 5, 6)

Interpretation: There is a no significant difference between size, location, lung zone, BS or VS class, NY and DY.

Interpretation: There is no significant difference in zone 3 between VS, BS and DY.

**Interpretation:**

1. Mean size of positive NY = 18,4 mm and negative NY = 13,2 mm: This is significant for the 10% level.

2. There is a significant difference (p=0.0018) between the bronchus sign classes for positive NY and negative NY.

Positive NY and BS class: 2aBS 100%, 2bBS 93%, 1BS 79% and 0BS 67%.

3. There is no significant difference (p=0,27) between vessel sign classes and NY.

Positive NY and VS class: 2aVS (PA) 89%, 2bVS 88%, 1VS (PV) 75% and 0VS 50%.

4. There is no significant difference between lung zones and NY.

5. There is no significant difference between positive or negative vessel sign resulting in a correct or false navigational yield within lung zone 3.

6. There is a significant difference (p=0,048) between a positive and negative bronchus sign resulting in a correct or false navigational yield within lung zone 3.

##### II) Extended univariate analysis for endobiopsy-group only

To test if there is a significant difference in both proportions for the combination of a favorable vessel sign class and an unfavorable bronchus sign class vs. crossover unfavorable vessel sign class and a favorable bronchus sign class.

A) 2aVS*(0-1)BS:

Diagnostic Yield: 22 TP / 1 FN

Navigational Yield: 17 TP / 6 FN

B) (0,1,2b)VS*(2a-2b)BS:

Diagnostic Yield: 13TP / 1FN

Navigational Yield: 13TP / 1FN

Fisher's Exact Test for both proportions:

P = 0,0074 (*univariate* analysis) for both proportions when you look at the above-mentioned combinations but no statistical difference for DY (alone) as mentioned in Table 3. This analysis hints toward the higher importance of a (positive) bronchus sign than of a positive vessel sign for outcome prediction.

Interpretation:

Navigational yield especially driven by rEBUS-positioning was much better when there was a favorable bronchus sign class - however DY was in both subgroups not significantly different and excellent (22 of 23 resp. 13 of 14).

##### III) Multivariate and multinominal logistic regression analysis of endobiopsy group (Table 7,8,9,10)

1) Odds of resulting in correct NY than in false NY will increase by 6.88 if moving from no BS (class 0BS) to any positive BS (p=0.007, 95% CI = 1.71-30.7)

2) Odds of resulting in correct NY than in false NY will increase by 22.00 if moving from class 0BS to class (2a+2b)BS = 2BS (p=0.006, 95% CI = 3.33 - 437.33)) and by 12.00 if moving from class 1BS to class 2BS (p=0.039, 95% CI = 1.39 - 256.10)

Interpretation: OR is more than 1 but the CI is very large so one cannot actually conclude much about the exact strength of this relation. However, class 2BS is one of the strongest factors for achieving correct navigational yield.

3) Odds of resulting in correct NY than in false NY will increase by 4.84 if moving from no BS (class 0BS) to a positive BS (classes 1,2a,2bBS) within lung zone 3 (p=0.043)

Interpretation: Any positive bronchus sign increases the probability of a correct navigational yield in comparison to the situation without any bronchus sign.

4) Odds of resulting in correct DY than in false DY will be reduced by 0.18 if moving from VS =PA (class 2aVS) to VS = PV (class 1VS) when class 0VS was extracted as control variable because of the 0 values. (p=0.182, 95% CI = 0.02 - 4.15, however not significant).

Interpretation: Although this finding is not significant it hints clearly to the probability that a pulmonary vein branch as vessel sign reduces for roughly factor 5 a correct diagnostic yield probability in comparison to a pulmonary artery branch as vessel sign. Therefore class 2aVS is much more important to predict correct diagnostic yield than class 1VS - in reality such vessel sign class could be rated as a clear factor not to go (easily) for an endobronchial approach.

5) Odds of resulting in correct DY than in false DY will increase by 3.18 if moving from no VS-PA (classes 0VS, 1VS, 2bVS) to a positive VS-PA (class 2aVS) within lung zone 3 (p=0.427, 95% CI = 0.12-85.09, however not significant)

Interpretation: Although this finding is not significant it hints clearly to the probability that a pulmonary artery branch as vessel sign increases roughly for factor 3 a correct DY probability in comparison to a situation without such pulmonary artery branch as vessel sign. Therefore class 2aVS is much more important to predict a correct diagnostic yield than any other vessel class.

6) Odds of resulting in correct DY than in false DY will increase by 1.61 if moving from no VS-PA (classes (0,1,2b)VS)) to VS-PA (2aVS) with Bronchus sign (binary) as control variable (p=0.615, 95% CI = 0.25 - 10.37, however not significant)

Interpretation: Although this finding is not significant it hints to the probability that a pulmonary artery branch as vessel sign increases roughly with a factor of 1.6 a correct diagnostic yield in comparison to a situation without such pulmonary artery branch as vessel sign even with a bronchus sign as reference. Therefore class 2aVS adds value in correct diagnostic yield prediction even when bronchus sign was already taken into consideration.

7) Odds of resulting in correct NY than in false NY will increase by 6.58 if moving from no BS (0BS) to a positive BS with vessel sign (binary) as control variable (p=0.010, 95% CI = 0.04 - 0.64)

8) Odds of resulting in correct NY than in false NY will increase by 7.20 if moving from no BS (0BS) to a positive BS with vessel sign PA (2aVS ) as control variable (p=0.007, 95% CI = 1.72 - 30.15)

**Interpretation**: When a vessel sign is already considered a positive bronchus sign renders roughly with a factor of 7 significantly additional value to predict correct navigational yield - this is even more true for a pulmonary artery branch as vessel sign (class 2aVS). In other words: The easiest iSPNs to reach endobronchially could be described with 2aVS*(1-2)BS especially 2aVS*2aBS.

### B) Additional statistics with partition models

A classic application of partitioning is to create in our study a diagnostic heuristic for a Tokoro classification (class of bronchus sign). Given some variables of interest (lung zone, size of tumors, class of bronchus sign and others) and outcomes (navigational yield beside others) for a number of subjects, partitioning can be used to generate a hierarchy of questions to help diagnose new patients. The Partition modeling recursively partitions data according to a relationship between the predictors and response values, creating a decision tree. The partition algorithm searches all possible splits of predictors to best predict the response. These splits (or partitions) of the data are done recursively to form a tree of decision rules. The splits continue until the desired fit is reached. The partition algorithm chooses optimum splits from a large number of possible splits, making it a powerful modeling, and data discovery tool.

The Partition platform recursively partitions data according to a relationship between the predictors (BS/VS, lung zone, 3D-size) and response values (e.g. NY(rEBUS)), creating a decision tree. The technique is often considered as a data mining technique for the following reasons:

- It is useful for exploring relationships without having a good prior model.
- It handles large problems easily.
- The results are interpretable.

In other words: Create a suitable data model by reorganizing outlier data points of the original raw data^12^-^14^.

The concept of tumor volume instead of mean size is the first step. Tumor volume expresses a better representation of an iSPN data and matches theoretically better the concept of Tokoro classification.

**Figure 3:**
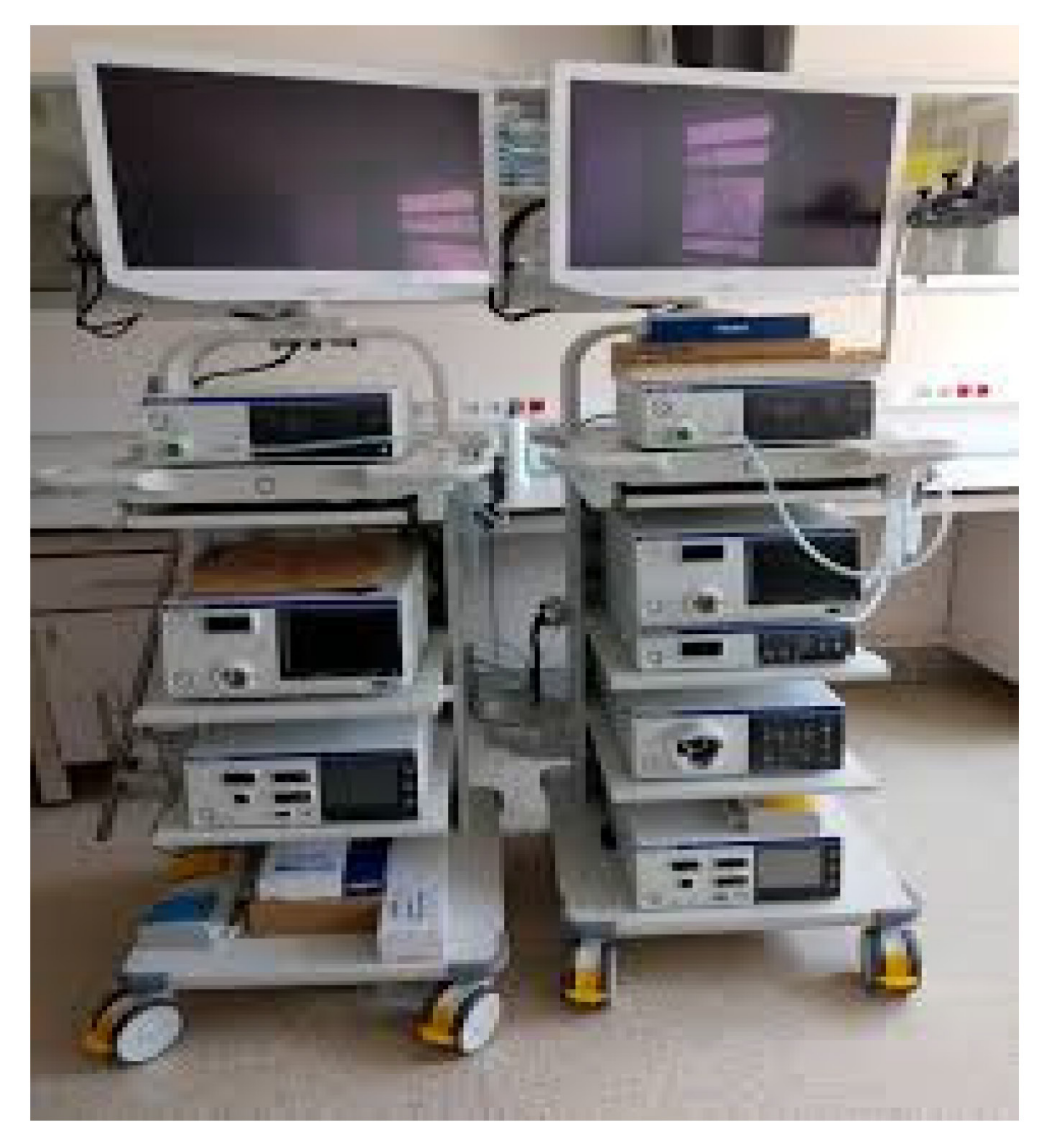
Olympus company (Olympus Europa SE & Co. KG, Hamburg, Germany). Exera III tower including all necessary processors and driving units for: BF-P190 (4,2/2mm), BF-Q190 (4,8/2mm), BF-P60 (4,9/2,2mm), BF-1TH190 (6,2/2,8mm) in combination with EWC (see below).

**Figure 4:**
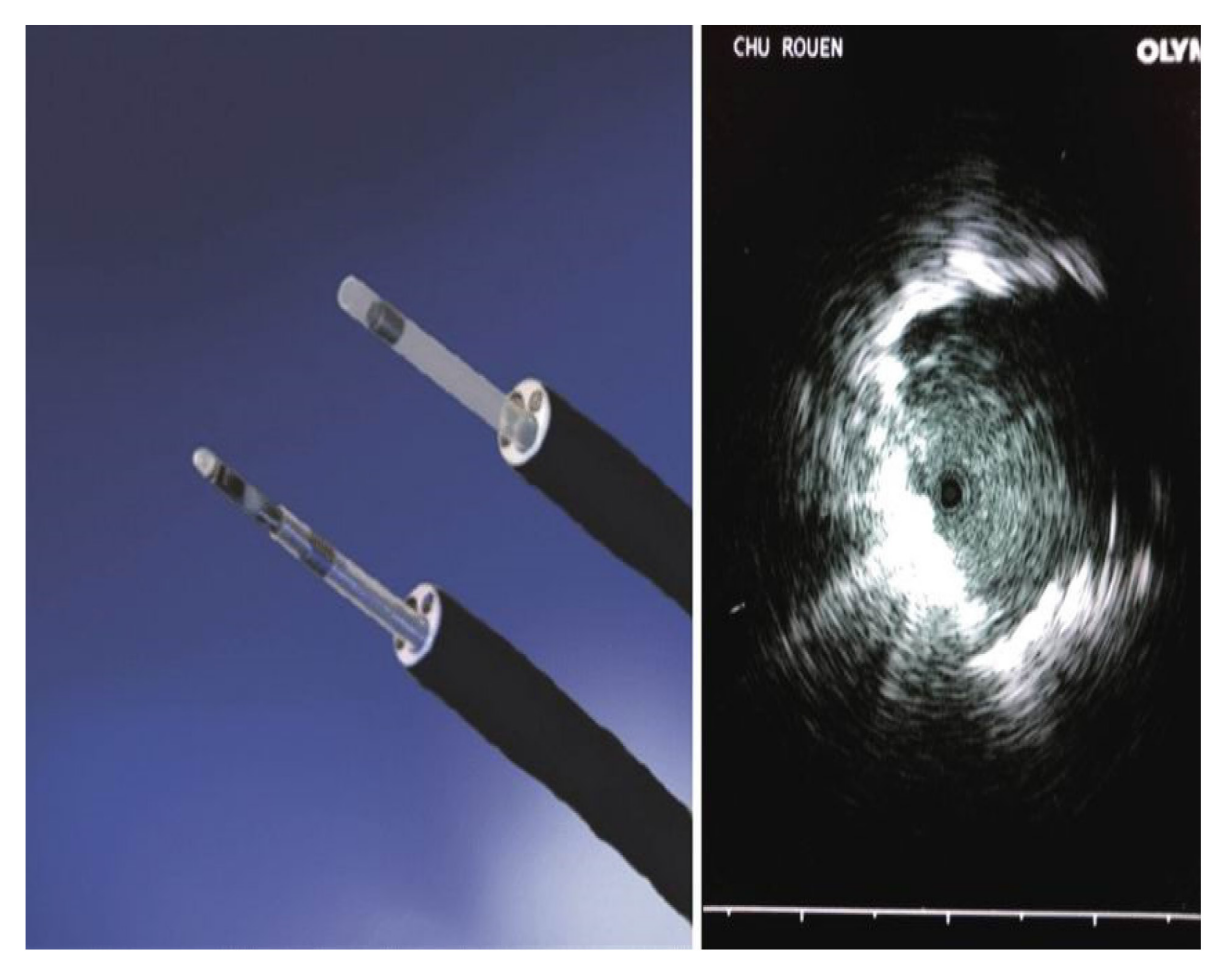
Radial ultrasound probe UM-S20-17S (1,4mm) and UM-S20-20R-3 (1,7mm w/o guide sheath).

Comparing the formulas of sphere [V=(W/2)^3*3,14*4/3^] and 3D-size [V=H*W*D] volume calculation their relationship to the applied mean size (H+W+D)/3 calculation in the raw data set gives an approximation (curvilinear trend, see Fig. 3) which is comparatively superior for 3D-size. Since both volumes describe themselves a very good linear relationship in log transformation (see Fig. 4), we used the 3D-size for further statistical purposes.

The relation between mean size in mm and 3D-size in mm^3^ is deduced by the equation:

a) Mean size = [3D-size]^1/3^ or

b) W=1.1994*(3D-size)^0.3155^

The concept of tumor volume instead of mean size is the first step. Tumor volume expresses a better representation of an iSPN data and matches theoretically better the concept of Tokoro classification.

Comparing the formulas of sphere [V=(W/2)^3*3,14*4/3^] and 3D-size [V=H*W*D] volume calculation their relationship to the applied mean size (H+W+D)/3 calculation in the raw data set gives an approximation (curvilinear trend, see Fig. 3) which is comparatively superior for 3D-size. Since both volumes describe themselves a very good linear relationship in log transformation (see Fig. 4), we used the 3D-size for further statistical purposes.

The relation between mean size in mm and 3D-size in mm^3^ is deduced by the equation:

a) Mean size = [3D-size]^1/3^ or

b) W=1.1994*(3D-size)^0.3155^

Table 13 displays the frequency distribution of the variables that potentially exert significant effects on the navigation response. The column BS-VS was created by concatenating the categories of bronchus sign and vessel sign. Five values were removed from the undecisive category (BS*2bVS) and allocated two to 2aPA category (2aBS*2aVS) and three to 2bPA (2bBS*2aVS) category. See reasons of rearrangement in the next paragraph. The categories 0No (0BS*0VS) and 2bNo (2bBS*0VS) are of minor importance, not influencing any statistical analysis performed.

**Figure 5:**
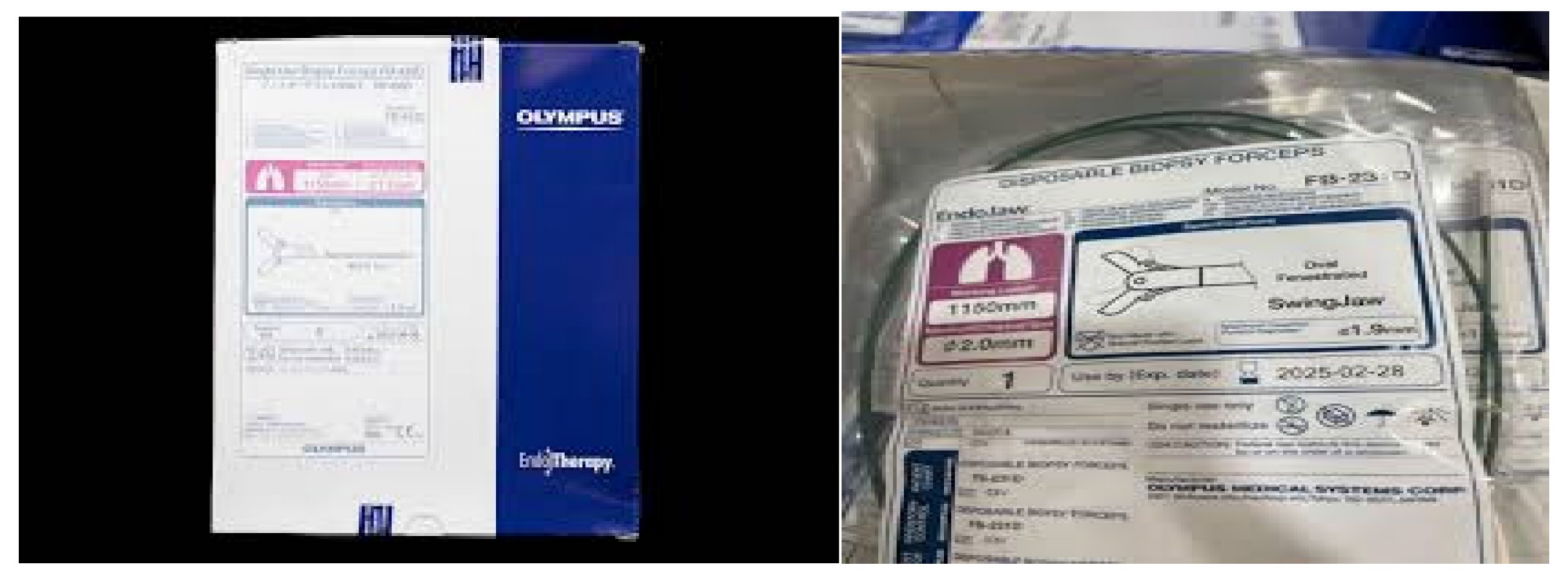
Various standard forceps sizes (including biopsy forceps FB-211D.A, FB-231D.A and FB-433D) and PeriView FLEX Needle NA-403D-2021), guide sheath kit (2mm), curette (2mm) CC-220DR Somatex company (Teltow, Germany) for 2 endobronchial needles: Broncho-Cut Expert 1,0 and 0,7.

The 3D-size of tumors was divided by the concatenated variable BS-VS, shown in Table 13, in groups and the output is depicted in figure 5. Obviously, the groups 1PA (1BS*2aVS) and 2bPA (2bBS*2aVS) share the highest values of 3D-size in accordance with some indecisive values from the same classifications as above and clearly a threshold of a 15000mm^3^ dimension could easily discriminates the whole data in two parts. Therefore, it was advised to allocate the colored extreme indecisive values to the corresponding Tokoro's classifications (2a and 2b) *(2aBS and 2bBS)*.

### 0BS*2aVS (0PA (Tokori's 0, PA presence) distribution and specific characteristics

Of particular importance are the categories OPA (0BS*2aVS), 1PA (1BS*2aVS), 2aPA (2aBS*2aVS) and 2bPA (2bBS*2aVS) in the column BS-VS, they form an affiliation between PA presence and the *(4 classes of)* bronchus signs [(BS-VS) 4] as depicted in Table 14 and further subdivided into two categories, OPA (Tokoro's 0 and PA presence) (0BS*2aVS) and BS^ (all the rest). The former is the key category of the study and is finally cross-tabulated with NY and lung zone to form the contingency table in Table 14. The latter corresponds to the positive values of bronchus sign (BS+, for NY=1).

According to OPA distribution in the contingency Table 14, four values were not identified (NY=0) in the zone 3, while 7 were positively yielded (NY=1) in the zone 3 and 2 in zone 2.

The size of tumors was particularly investigated for possible effects of the Tokoro classifications by conducting a one-way ANOVA in log10 transformation to bring values to normality. The results are depicted in Figure 12, in which there exists a significant effect of Tokoro locations (p=0.005) and that is further analyzed by plotting the least square mean 3D-sizes across the Tokoro categories and also applying the Fisher's LSD test of pairwise mean comparisons. The two types of analysis show that OPA (0BS*2aVS) and 1PA (1BS*2aVS) are expressed equally by lower tumor mean sizes as compared to the higher ones of 2bPA (2bBS*2aVS) and 2aPA (2aBS*2aVS) being equal each other: 0PA = 1PA < 2aPA = 2bPA. (0BS*2aVS = 1BS*2aVS < 2aBS*2aVS = 2bBS*2aVS) This inference comes out because the two groups share different letters and also the 95% confidence intervals of the two group mean hardly overlap. Thus, small tumors are expected in areas with Tokoro codes 0 *(0BS)* and 1 *(1BS)* in joint with PA presence *(2aVS)*. And the smaller size perhaps eludes sometimes the positive detection (NY=1).

Indeed, the NY fails to detect efficiently smaller tumor sizes Figure 13, particulary in the OPA (0BS*2aVS) category (69,2%), provides better recovery in the 1PA (1BS*2aVS) category (80%) and complete success in the two other categories (100%, 2aPA and 2bPA) (100%, 2aBS*2aVS and 2bBS*2aVS).

In order to isolate the OPA (0BS*2aVS) performance from the rest 3 Tokoro-PA (BS*2aVS) combinations (combined BS^) on the NY response including also the lung zone and the 3D-size, as all demonstrated in the cross-tabulation of Table 14, a specific statistical technique of data partition in groups (nodes) was employed.

In our study, the partition platform recursively partitions data according to a relationship between the **predictors BSVS, lung zone, 3D-size** and the **response** variable (**NY(rEBUS**)), creating first a small decision tree (Exhibit 3). All data are primarily split via BSVS in two parts, BS^ and 0PA (0BS*2aVS), followed by lung division on either side into the zones 2 and 3, then by particular splitting cut values of 3D-size ending to various nodes and finally terminating to the 0PA (0BS*2aVS) node at a cut value of 3D-size≥1287 mm^3^.

Thus, the 0PA (0BS*2aVS) category can be driven by a partition algorithm following the route:

All data→BSVS→0PA→lung zone 3→3D-size≥1287→0PA node

The decision tree is finally described by a R-square value of 0,358 of the total variation of data including 77 values and creating 7 data splits leading to 7 nodes as depicted in Exhibit 3.

Since the response is categorical (NY), the fitted value is a probability for the levels of the response and the split is chosen to minimize the residual log-likelihood chi-square (see the manual pdf for further details). The criterion **G^2** is a fit statistic used for categorical and lower values indicate a better fit. The **Logworth** statistic is defined as -log10(p-value) and the optimal split is the one that maximizes the logworth. The S**plit History** shows a plot of RSquare versus the number of splits chosen for the study towards to an asymptote direction. The **Column Contributions** shows a report indicating each input column's contribution to the fit and how many times it defined a split and the total G^2 attributed to that column. Thus, BSVS is the most important variable for the study explained 40,64% of the G^2 statistic using 2 splits, followed by the tumor 3D-size sharing 39,64% and 3 splits and finally by the lung zone (19,72% and 2 splits). The **Leaf Report** shows the mean and count or rates for the bottom-level leaves of the report and the colored leaves mark the 0PA counts and probabilities totaling 13 probable 0PA counts and reflecting three nodes of interest in the decision tree:

The BSVS (0PA) node includes 8 0PA (0BS*2aVS) counts with 87% NY efficiency and one patient tumor escaping from detection. Thus, 7 0PA (0BS*2aVS) counts were detected in zone 3 with 3D-size ≥1287 mm^3^ and concern the following patients' characteristics:

The node defined by 3D-size<1287 mm^3^ includes 3 0PA (0BS*2aVS) counts not detected in zone 3 below 1287 mm^3^ tumor sizes (NY=0), so greatly contributing to the reduction of NY efficiency down to 47,32% and essentially revealing the failure of the NY(rEBUS) equipment to succeed in tumors with such 0PA (0BS*2aVS) low volumes.

In the lung zone 2, in contrast to the above size limitation, 2 0PA counts were detected with 3D-size < 1078 mm^3^ (see the node defined by 3D-size<1078). The NY efficiency reached 81%, that is the technique succeed in 4/5 counts but failed to produce a node with pure 0PA (0BS*2aVS) counts.

To assemble the findings so far, a prediction profiler was interactively built setting specific values of the three predictors on NY response. Changes in the predictor values are reflected in the estimated classification probabilities. Setting the 3D-size at the two values of high interest (1078 and 1287mm^3^) it comes out that the predicted NY efficiency approaches nearly 100% (97,9%) in zone 2 no matter the size of the tumor and, in zone 3, 87% in cases where the 3D-size is ≥1287mm^3^ and 47,3% when the 3D-size is <1278mm^3^ and even <1078mm^3^, failing completely to detect the tumors (NY=0).

#### Prediction profiler

In terms of clinical characteristics in the diagnostic yield of endobioscopy the findings were sorted according to a confusion matrix and a ROC curve to the following results:

The** Confusion Matrix** is a two-way classification of actual and predicted responses and the present partition analysis produces the 2x2 contingency table by numbers and rates.

**The Receiver Operating Characteristic (ROC**) curve displays the efficiency of the model's fitted probabilities to sort the response levels of NY. Judging from the track of the two lines, the distance from the diagonal line (equal to 50% rejection) and mostly from the high AUC value (0,895), it follows that the model performs very adequately and could be safely used for new entries of patient records to predict, in high probability, correctly the NY values.

It is noteworthy, that the decision tree of the study achieves to detect the 13 0PA (0BS*2aVS) counts in three discrete nodes with specific attributes each (Supplementary file).

The described partition model derives a significant (p=0,0052) vessel sign model Figure 12. The probability of correct navigational yield is for an iSPN > 1287mm^3^ (mean size >11mm) in zone 3 69% for the description 0BS*2aVS.

In univariate analysis (see Table 5) probability for the following classes were:

BS class and positive NY: 0BS 67% (p=0.0018)

VS class and positive NY: 0VS 50% (p=0.27, therefore not significant)

#### Analysis on transbronchial access technique (TBAT) in the endobiopsy group

This technique is aggressive to the parenchyma, time consuming and costly depending on the used tools. It was only allowed in the endobiopsy group especially to ameliorate the intratumoral positioning of our biopsy instrument. Following the hypothesis of our study iSPNs performed with TBAT revealed very often 2aVS (PA-branch) and its frequency were depending on the given BS class:

13 of 15 TBATs in zone 3 revealed a class 2aVS.

5 of 13 (38,5%) iSPNs presented with 0BS*2aVS.

2 of 10 (20%) iSPNs presented with 1BS*2aVS.

3 of 12 (25%) iSPNs presented with 2bBS*2aVS

3 of 24 (12,5%) iSPNs presented with 2aBS*2aVS.

In total we applied 21 TBATs (27%) in zone 2 and 3 in 77 lesions in the endobiopsy group.

**Figure 9:**
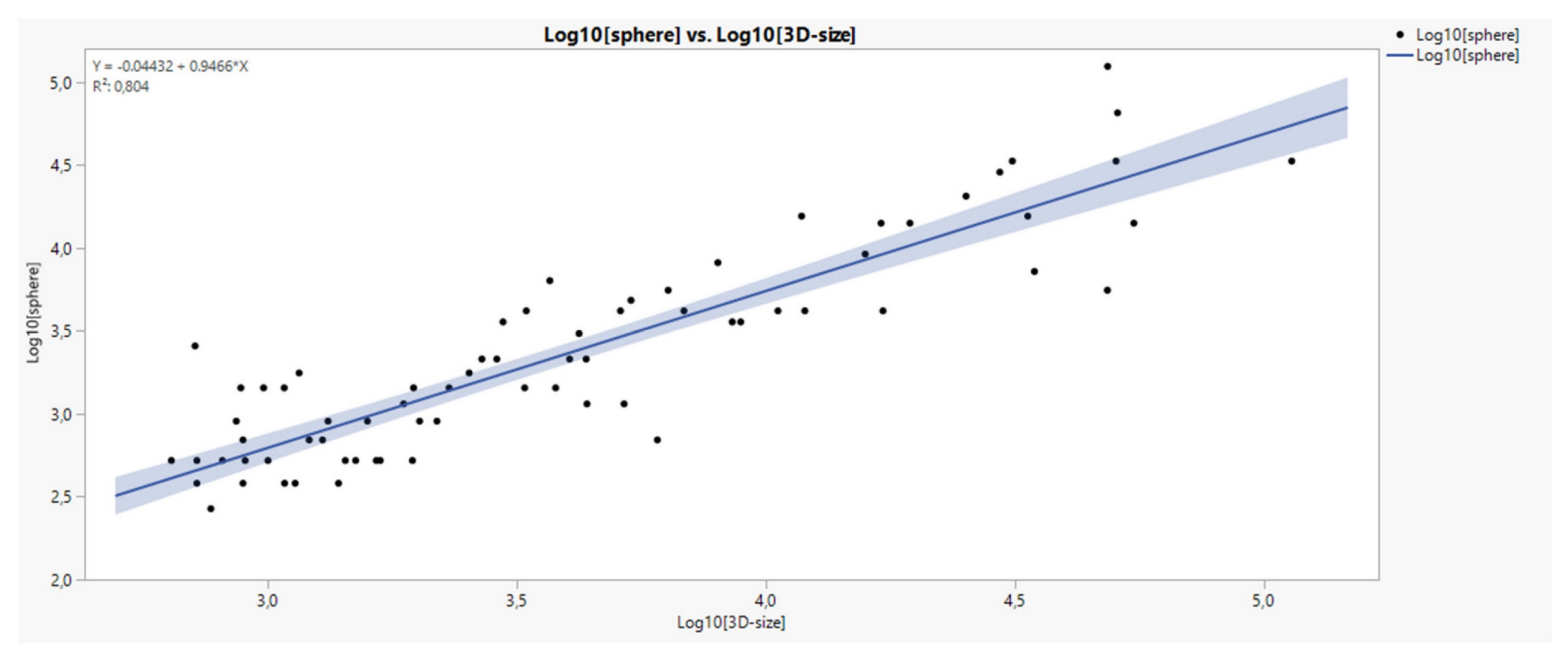
Linear relationship between sphere and 3D-size in log transformation.

**Figure 12:**
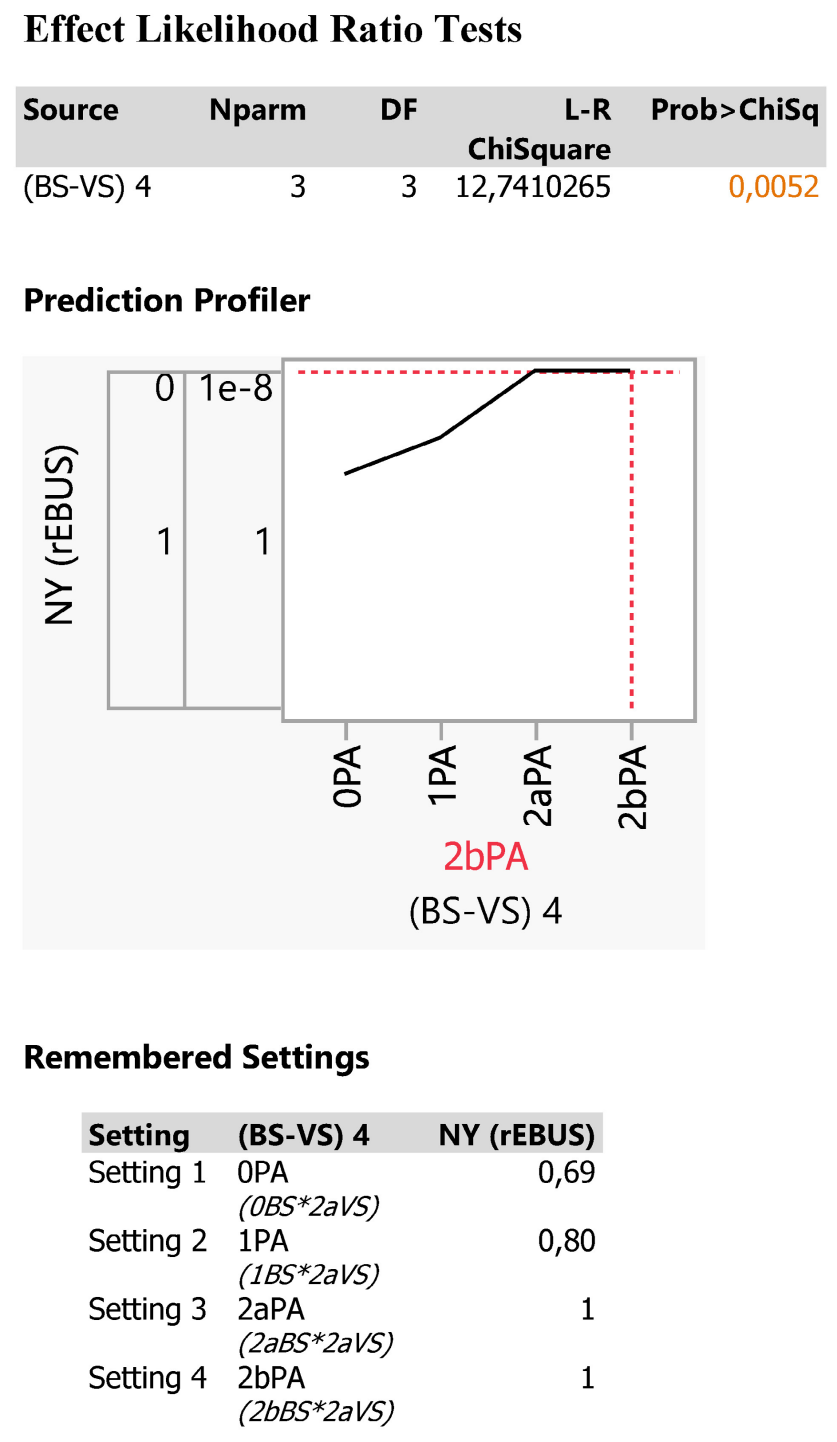
Statistical report of the response NY as affected by the 4 Tokoro-PA combinations. The effect is statistically significant at p=0,0052 probability level.

**Figure 13:**
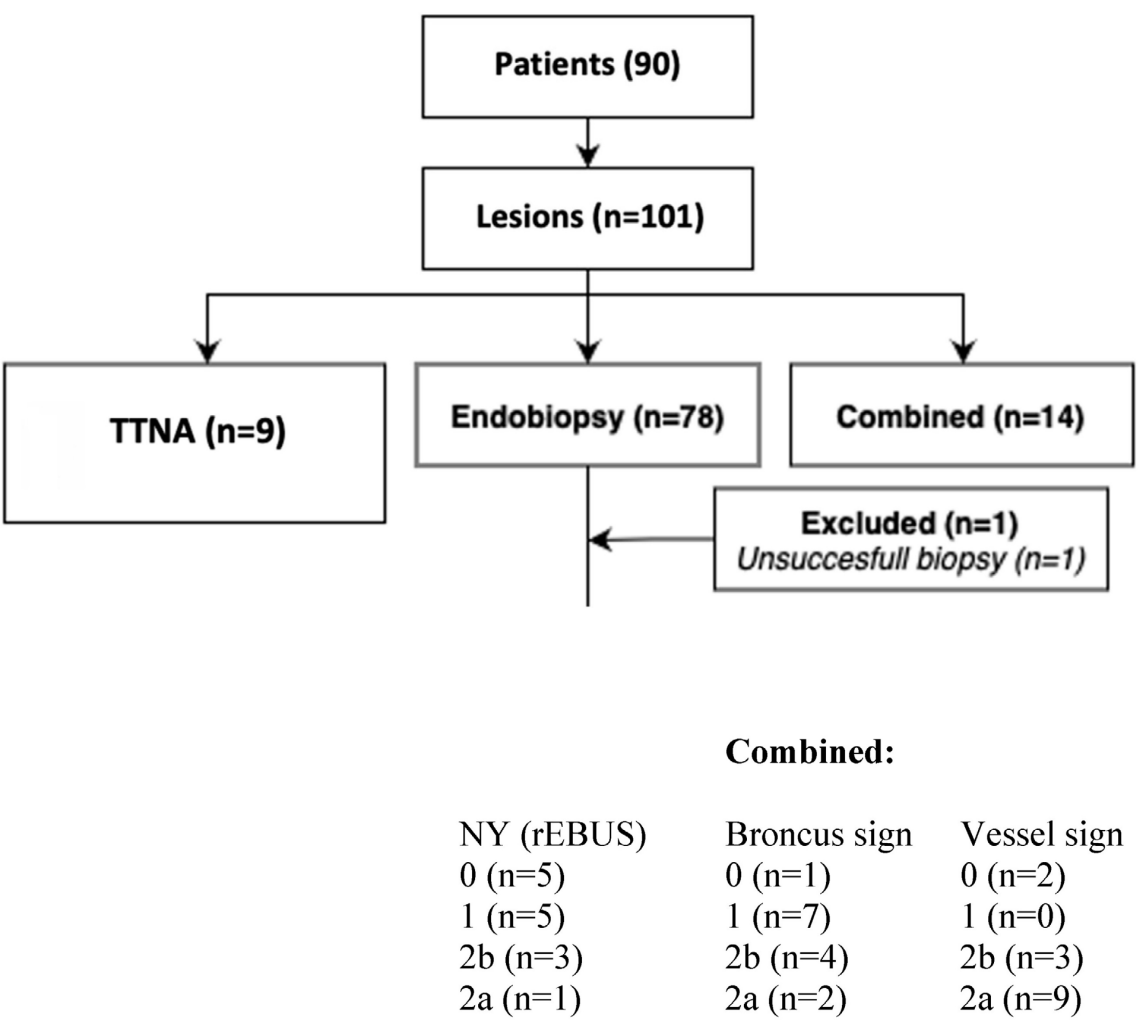
Combined and TTNA group, whole study population.

One has to remember (see Fig. 9) that in regards to correct navigational yield (rEBUS) we missed 10 of 77 (13%) whereas in regards to correct diagnostic yield we only missed 5 of 77 (6,5%). In older navigational literature (with rEBUS alone) the relation of NY and DY is exactly the opposite with roughly minus 20% for DY vs NY^15^.

The reason for this phenomenon is in our understanding clearly the combination of TBAT and AF: AF was always the final step to decide whether to biopsy in the reached position or to optimize again.

#### TTNA- and COMBINED group, the whole group and others

We did not miss any iSPN in the both groups - pure TTNA and COMBINED. All iSPNs in the COMBINED group (and in the pure TTNA group) were finally biopsied with a transthoracical approach, representing 23% of all approaches.

**Interpretation:** The iSPNs of the endobiopsy group (median 14mm) were significantly smaller than the iSPNs of the rest of the study group (median 25mm).

**Interpretation**: In the 2 different regression models the TTNA+ COMBINED groups had a significantly unfavourable vessel sign pattern in comparison to the endobiopsy group which seems to be as well true for the bronchus sign pattern (Table 19). This result reflects the general hypothesis and the individual decision of the examiners that a positive vessel sign (especially class 2) is a clear argument to go for endobronchial approach.

**Interpretation:** XR visibility was significantly depending on size with roughly 12mm as in general invisible size cut-off.

In 100 lesions predefined modalities pure endobiopsy, pure TTNA and combined approaches were performed in 77, 9 and 14 lesions respectively. In these 3 modalities we found confirmed (mostly specific) benign and malignant cases 47 and 30, 4 and 5, 2 and 12 respectively. Lesion sizes in the 3 different groups were (median, mean) 14 and 17,7mm (of those 41 invisible of 77 under XR (53%) in the endobiopsy group), 27 and 31mm, 18,5 and 23mm respectively. In the 3 groups for the malignant cases 25 of 30 (83,3%), 5 of 5 and 12 of 12 were diagnosed correctly rendering a diagnostic yield of 42 in 47 malignant cases for the whole algorithm (89,4%) with sizes (mean, median) for the whole algorithm of 16 and 19,7mm respectively. In regards to vessel sign analysis it has to be clearly stated that the significance level for outcome prediction is inferior to bronchus sign analysis. In multivariate analysis there is a clear tendency towards higher outcome prediction especially if a pulmonary artery branch leads into such target even when a bronchus sign is missing. For NY when comparing univariate analysis and partition model analysis at a set diameter of >11mm and in zone 3 the additional advantage of analysing a given pulmonary arterial branch vessel sign seems to add on 19% of correct outcome prediction for NY with significance (p=0,0052) even when a bronchus sign is missing (0BS*2aVS: 69% correct NY)^16^.

## Discussion

To the best of our knowledge this paper describes as first-in-men the optimized use of a COMBINED algorithm along vessel sign analysis. Furthermore it is to the best of our knowledge the first-in-men paper covering a detailed analysis of the value of vessel sign in a prospective 3-digit registry. Due to the fact that especially Interventional Radiologists have a vast experience in intervening in a CT or CBCT environment we as Interventional Pulmonology group already used to go transthoracic by ultrasound for pleura-attached consolidations or by CT or XR for deeper lesions. We asked ourselves how to optimize the CBCT application in order to increase DY, to reduce time on-table (limited to be less than 1 hour in all cases) and to reduce complications by using different modalities: The hereby presented algorithm includes 3 CBCT driven modalities with on basis of the systematic analysis of the preset-CT mainly preinterventionally decided between pure endobronchial, pure transthoracic or a so-called combined approach. Anatomy teaches us as a matter of fact that vascular tree (pulmonary artery (PA), pulmonary vein (PV) and lymphatic vessels) as well as nerves follow in zone 1 and 2 aside the bronchus - the so called bronchopulmonary trias (BPT) - whereas in zone 3 typically representing the subpleural or pleura-attached lung area a pulmonary artery branch is in the centre of the secondary lobule (SL) and the outer border of this unique anatomical lung parenchyma structure is defined by pulmonary vein branches and lymphatic vessels.

At the same time the content of cartilage is so far reduced in zone 3 in comparison to zone 1 or 2 that our group tends to speak in respect to this zone 3 about a 'cheesecake situation': Our endoscopical instruments follow tubular structures which in zone 3 show less resistance due to more and more gaps in the cartilage continuum until zero cartilage in comparison to zone 1 and 2. This situation eases the (relatively) unpredictable maneuverability of different instruments which is still the missing link to perfect positioning of instruments in a targeted iSPN (e.g. centered positioning is aspired except for cystic malignant lesions) even with pre-bended edgecaths or newer active stearable sheaths.

In regards to anatomy of the bronchial tree having beyond the 15^th^ segmentation nearly no cartilage as wall structure it could be translated as the entry point into the SL which is situated with a depth of 2,5 to 3,5 cm below visceral pleura in horizontal CT-slices and showing an estimated width around 2 to 3cm in perpendicular to the longitudinal axis. According to anatomy at this entry point pulmonary vein branches are deviated aside from the longitudinal axis of a secondary lobule forming the borders along with lymphatic vessels whereas the pulmonary artery branches will follow this longitudinal axis into the center of a SL. This axis can be interpreted as the prolongation of the BPT of zone 2 into zone 3. In other words: Such situation renders in a navigational environment an expected pulmonary artery branch bulls-eye view even when a bronchus sign is missing. PA+VS and negative BS situation has shown in a retrospective analysis of Ho *et al.*^17^ of 30 iSPNs of an EMN registry a very high diagnostic yield of 96% although not mentioning other factors.

Amid this above-mentioned missing link of peripheral outcome prediction 3-dimensional external near real-time navigation by CBCT segmentation of targets and paths helps Interventional Pulmonologists to achieve more sensitive positions with classical (yet unexpensive) instruments like small bronchoscopes (and in case they are missing the use of EWC), sheaths, needles, brushes, curettes and forceps in a shorter time than without CBCT. However going 'cross country' and leaving the predefined tubular path is an option but by far more time and expenses consuming approach to reach iSPNs at the border of a SL whereas as alternative a transthoracic approach in experienced hands is a fast, safe and cheap procedure although one buys this option with a higher rate of pneumothorax (and bleeding) risks in comparison to endobronchial approach^18^. Of note is the fact that with transbronchial access (e.g. tunneling device) there is as well an increased risk to destroy the predefined anatomy with the consequence of focal small pneumothoraces (destroying microanatomy and harming the endobronchial navigation) or peritumoral bleeding reducing the intraoperative visualization options which could lead to further impossible-to-perform situations. CT and especially CBCT visualization depends on contrast between target and surrounding parenchyma. One possibility is hyperinflation of a navigated area as air gives a good black-contrast. However, hyperinflation in the field of CBCT application increases the risk of pneumothorax due to increased mean airway pressure and reduce possibly hemodynamics due to patients' comorbidity especially in right heart deficiency often seen in chronic lung diseases. It may increase air embolism especially when the patient is positioned laterally and the iSPN is above right atrium level. It may increase paCO2 over time on table as effective ventilation with a modest relation between inspiration and expiration is not applied in hyperinflation mode: We use long lasting inspiratory flows (1/min, I:E 3:1) with relatively highly titrated working pressures for segmentation and navigation^10^. In periods without navigation purpose normal ventilation is applied.

Interestingly we found no such hint in our paper about nasal jet-catheter ventilation although a clear tendency over time (especially after 47 min) to increase paCO2 significantly with nasal jet-catheter ventilation on intermittent hyperinflation mode as described above^8^. Of note is the fact that the most influencing variable for an elevated paCO2 during the intervention was the paCO2 before the intervention.

Another way to increase contrast in a predominant aerated parenchyma is to apply vascular contrast media agents if the target consists partly of vessels^19^. As this technique is not a regular option for the most Interventional Pulmonologists during an intervention it is of special interest that small bronchiole due to the small thickness of walls are in general not detectable by CT technology in zone 3 (especially without hyperinflation) and even beyond the 8^th^ segmentation) which is clearly in zone 2 - and this in opposite is not true for blood perfused vessels with a good naive white contrast^20^. In 2015 we have already studied (but not published) in 50 patients on a CBCT platform this idea with an (essentially different than below) definition of 'combined approach': It was the decision of the examiner in that modality to decide on endobronchial and transthoracic biopsy (meaning always 2 biopsies) for the same iSPN during the same examination with the result that a DY of 90% was achieved on the cost of 25% pneumothorax (3 of 12 cases) in this group whereas the pneumothorax rate in the pure transthoracic approach or the pure endobronchial approach was 0% (0 in 4 cases) resp. below 3% (1 in 34 cases). Therefore we believe that simultaneous biopsying an iSPN by endobronchial and transthoracic means during the same examination renders to high risks so that we have left that concept and introduce the above mentioned different algorithm.

## Conclusion

A nodule orientated approach in a manual CBCT-AF environment including typical instruments renders in experienced hands comparable results to robotic assisted bronchoscopy. In multivariate analysis only bronchus sign analysis revealed significant (p = 0,05) prediction of navigational yield outcome prediction whereas vessel sign analysis increases highly the odds ratio in favor of positive outcome prediction but without significance at the given level.

In reference to this paper one can conclude the following assumptions:

1. A favorable bronchus sign offers a significant positive outcome prediction. This result was highly significant in univariate and multivariate statistical analysis especially for navigational yield.

2. A favorable bronchus sign according to multinominal regression models offers at least a 4 times stronger factor for positive outcome prediction (this is: Correct diagnostic yield for pulmonary arterial branch and correct navigational yield for bronchus sign) than a pulmonary artery branch vessel sign. (Table 11 and 12).

3. However, a favorable pulmonary artery branch vessel sign increases in any case the probability of a correct navigational yield which seems to be additional 19% correct outcome prediction for navigational yield even when a bronchus sign is missing comparing results of univariate and partition model analysis (see Results IV: Partition model and Table 5).

4. A favorable pulmonary artery branch as vessel sign seems (result not significant) to increase roughly with a factor of 3 the outcome prediction for a correct navigational yield comparing to a situation without such class of vessel sign (Table 10).

5. In contrast a pulmonary vein as vessel sign seems (result not significant) to decrease with a factor of 5 the outcome prediction of a correct navigational yield in comparison to a pulmonary artery sign as vessel sign. This result reflects anatomy of the secondary lobule in zone 3 (Table 9).

6. A missing bronchus sign seems (result not significant) to increase the essential need for more devices (roughly 3times higher frequency of TBAT) in comparison to a bronchus sign leading into the center of the target to reach a positive navigational yield for all targets in which a pulmonary artery branch was present (Results V: TBAT analysis).

7. Augmented fluoroscopy analysis as the final step (after rEBUS driven navigation and) before a biopsy decision as a unique property of an even manual CBCT environment paired with a high expertise rendered a higher positive diagnostic yield ratio (72 of 77 targets; 93,5%) than the navigational yield ratio (67 of 77; 87%) in the endobiopsy group with diameters below 20mm. This finding is in contrast to former navigational rEBUS only environments without CBCT-AF in which navigational yield was on average 20% higher than diagnostic yield^15^.

8. A combined approach - leaving a bronchoscope in a navigated position before transthoracic biopsy - seems to reduce adverse events as we had zero negative events in this subgroup.

9. Using all possible modalities in a manual CBCT environment allowed us to perform 100 biopsies in always less than one hour as time-on-examination rendering a correct diagnostic yield of 95% (all dignities) and in 42 of 47 malignant cases for the whole study group (89,4%) with sizes (mean, median) for the whole study group of 16 and 19,7mm respectively. Malignancy was correctly diagnosed in 83% in the endobiopsy group which provided 30 of 47 malignant cases (64%) of the whole malignant study population. All these results are comparable and non-inferior to recently published results of robotic assisted bronchoscopy (RAB)^21^-^23^.

10. Pulmonary CBCT navigational interventions can be performed without the help of anesthesiologists in experienced hands with profound knowledge of the interaction of different ventilation modes and hemodynamics.

Translating these assumptions into the field of therapeutic interventions under CBCT guidance one should respect the idea that not every malignant nodule is easily suitable for such approach in the sense of time limitation and easiness of approach: Success of modern radiotherapy, minimal-invasive surgery, targeted systemic therapy even in stage I lung cancers and especially the combination of all these modalities imply just now an abundance of more or less positive data so that interventional pulmonologists with the idea to treat endobronchially are pretty late on the competitive field of therapeutical options. In other words: We should not waste too much energy to go for every malignant nodule but we should determine carefully which lung nodules promise good success. This paper paves a way for the best selection of such an approach.

This paper shows some weakness:

It is noteworthy that all examiners had a vast experience in CBCT application, especially the first author has performed by far more than 1000 on different machines. Therefore these results cannot be translated into every CBCT environment. Furthermore, we have not performed this study with the help of ultrathin bronchoscopes and especially not with ROSE on table. As mentioned above with ROSE are diagnostic yields of 85% reported even without CBCT. We have not applied CBCT-TIL which renders according to actual literature another 12% of diagnostic yield on the costs of radiation dosage and time-on-examination. There are ongoing discussions about specific histology results which may reduce diagnostic yield especially in the benign lesions group. However, this factor is influenced especially by the pathologists and not by the interventionalists. In our study this would have reduced positive diagnostic yield in 15 cases for benign disease but not for malignant disease: For the whole study population the DY would have been still 80% which is in the range of RAB diagnostic yield. As this difference did not change the clinical course of all patients we still assume the mentioned yields as the correct and relevant ones. In regards to 23% of transthoracic approaches instead of endobronchial approaches one can argue that this paper is not a pure Interventional Pulmonology paper. In our understanding Interventional Pulmonology covered for decades as well the possibility to solve diagnostical problems transthoracically. As this paper is about the optimization of CBCT application we still believe that this part of the registry is accepted as a performance in Interventional Pulmonology which has clear overlap with Interventional Radiology. Our algorithm is nodule (and therefore patient) orientated - nor technique neither department orientated. However, there are first hints in the literature that even in early lung cancer (stage I) a transthoracic approach could lead to an earlier relapse in comparison to an endobronchial approach^24^. The first 3-digit prospective registry about vessels sign analysis did not show significant results about vessel sign anatomy. But it did show a clear tendency supporting the above-mentioned hypothesis that a pulmonary artery branch as vessel sign (class 2aVS) has a higher value for positive outcome prediction than any other vessel sign class. The reason for this finding without significance could be the size of the dataset and the anatomy itself. Firstly, we had yet 17 of 77 (22%) targets in the endobiopsy group with vessels of unclear origin (e.g. branches of pulmonary artery or vein). Secondly, the anatomy description of BPT has to be understood in the way that the relative localization of pulmonary artery and vein branches around the accompanied bronchus may change (e.g. vessels changing the side of the bronchus along the same bronchus) following a hilofugal pattern. That said a pulmonary artery branch understood as the entry point to a SL could be 'misplaced' in comparison to an ideal anatomy part of our hypothesis for expected 2-4 mm. This interpretation is supported by the finding that the significant partition model (in regards to 4 Tokoro classifications*2aVS) analysis revealed a higher diagnostic yield in smaller nodules in zone 2 than in zone 3: The partition model with an ROC AUC of 0,89 for rEBUS NY derived a significant NY for 0BS*2aVS class at a given diameter of at least 11mm in zone 3 with 0,69 efficiency whereas in zone 2 for nodules <11mm of the same class the NY efficiency was 0,81.

## Supplementary Material

Supplementary information.

## Figures and Tables

**Figure 1:**
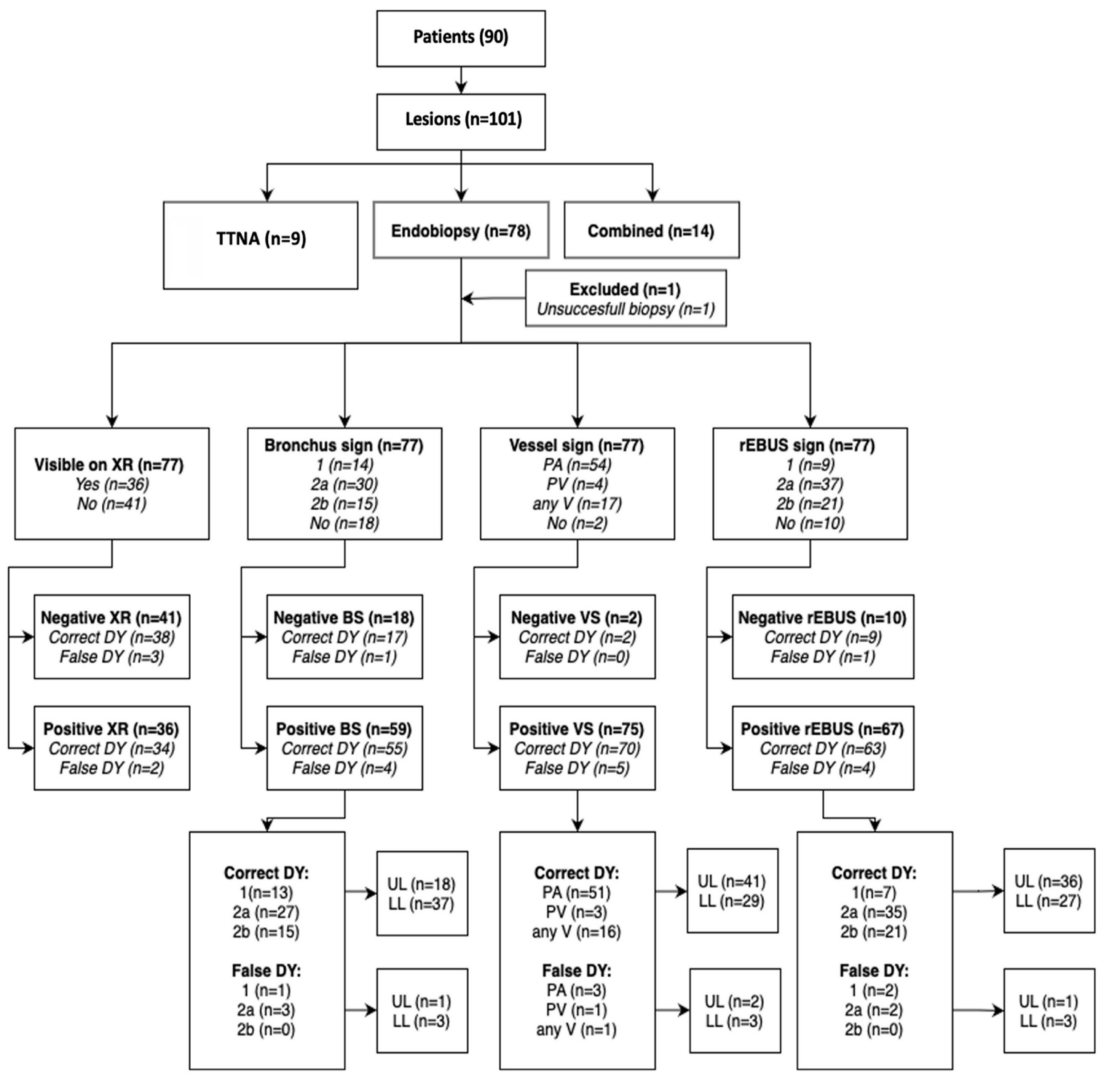
Workflow of the COMBINED algorithm.

**Figure 2:**
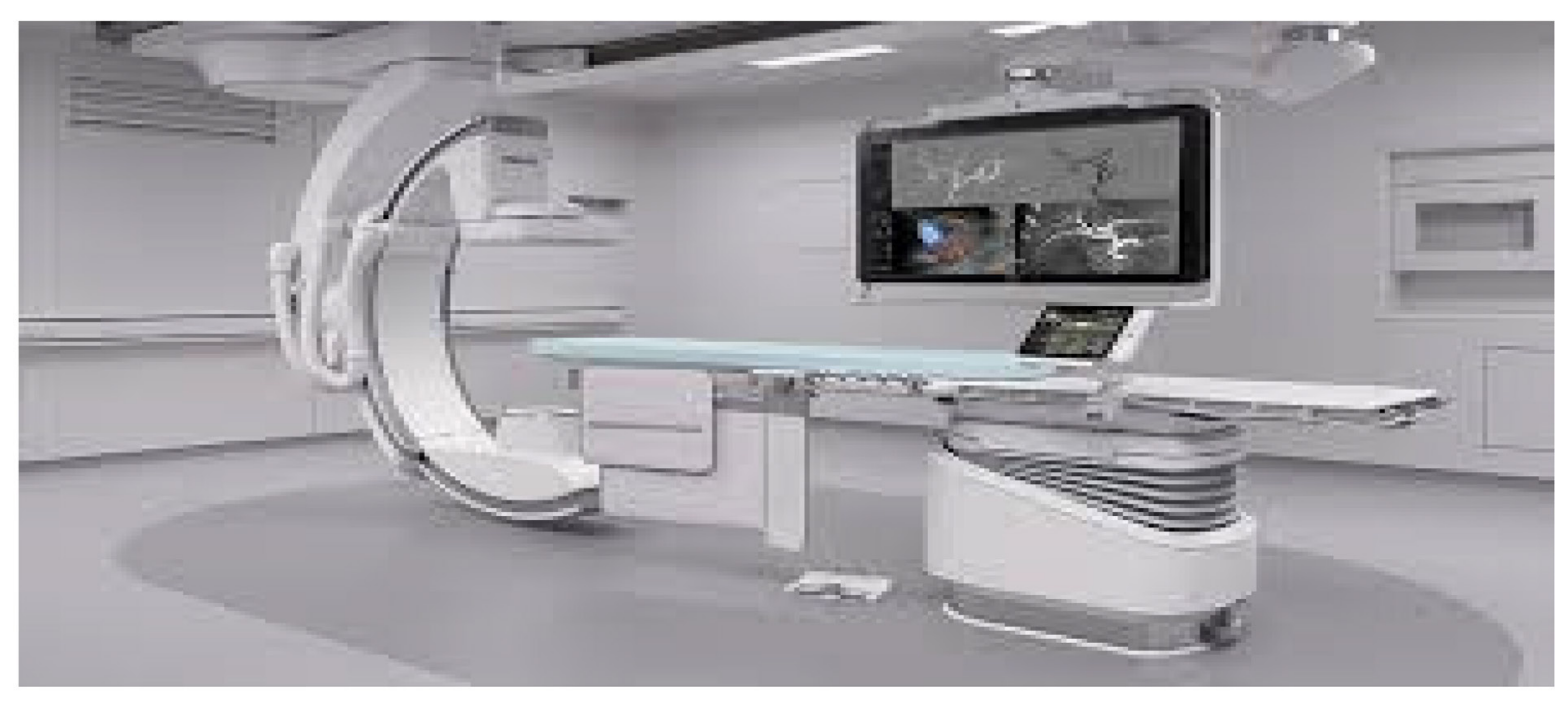
CBCT Azurion 7.1. (ceiling mounted) from Philips company (Koninklijke Philips N.V., Amsterdam, The Netherlands).

**Figure 6:**
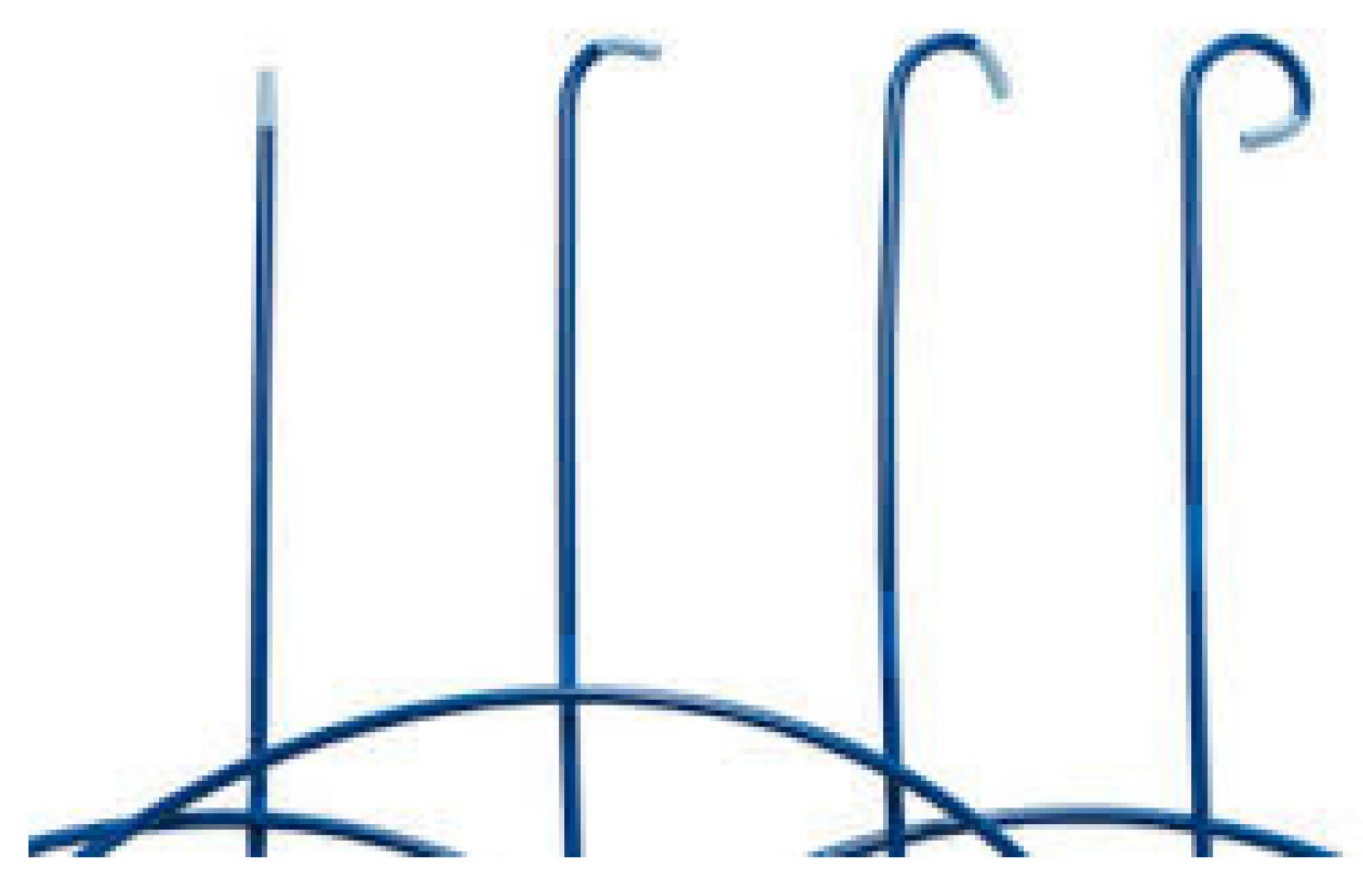
Medtronic company (Meerbusch, Germany). Extended working channel (EWC) SD180EWCTE-FT (2,68/2,08mm).

**Figure 7:**
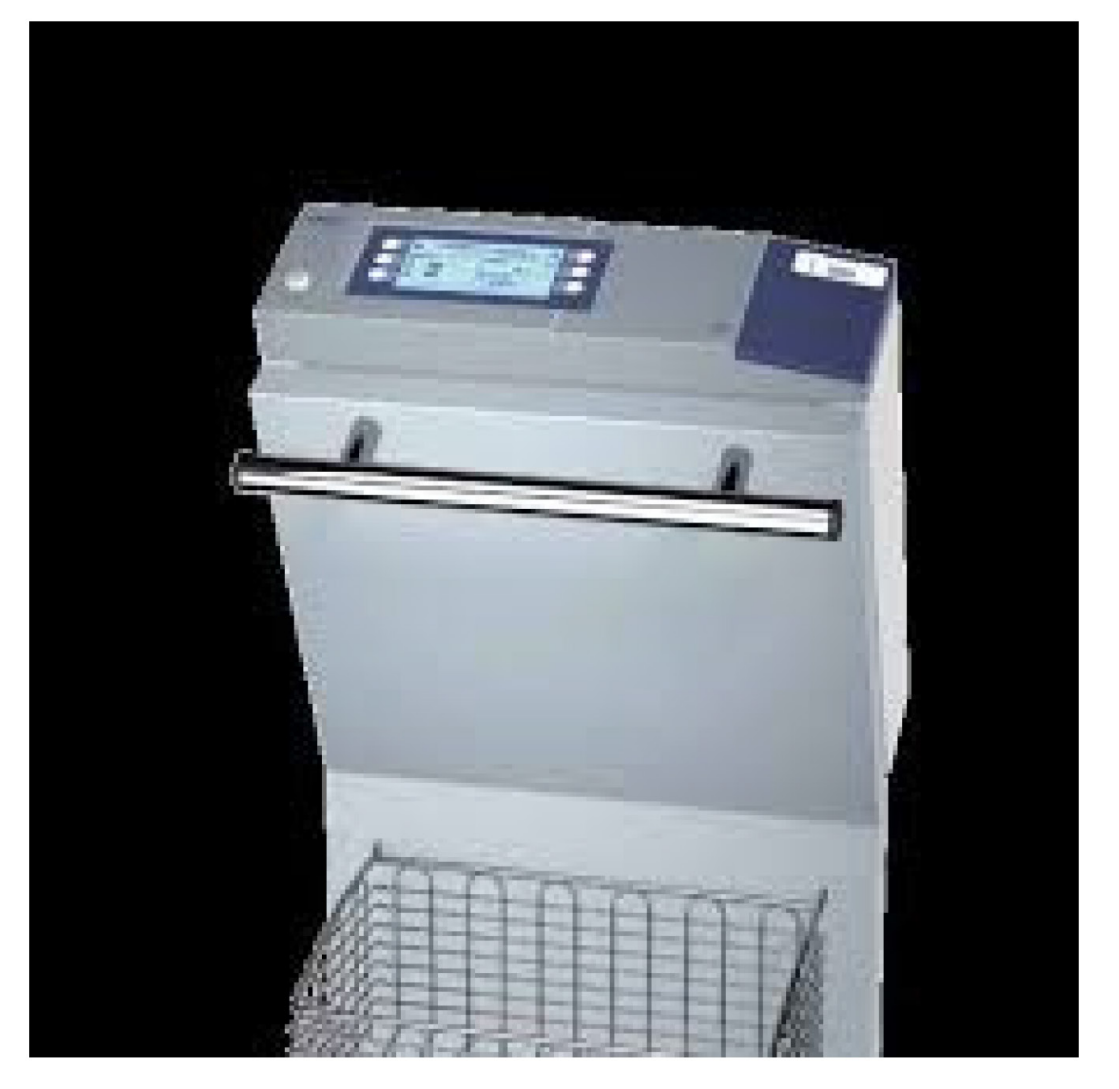
ERBE-CRYO II cryo generator with single use cryoprobes w/o sheath 1,1 and 1,7mm.

**Figure 8:**
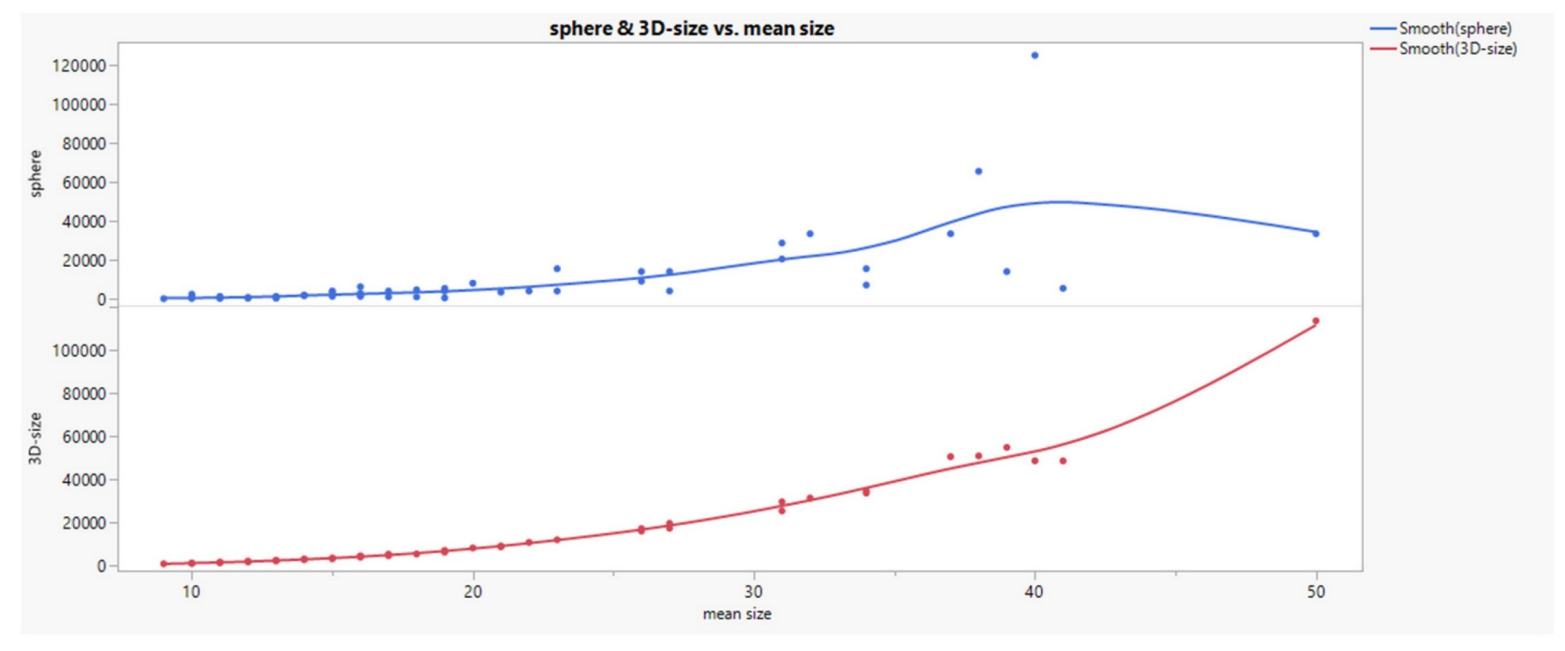
Relationships between sphere and 3D-size vs mean size.

**Figure 10:**
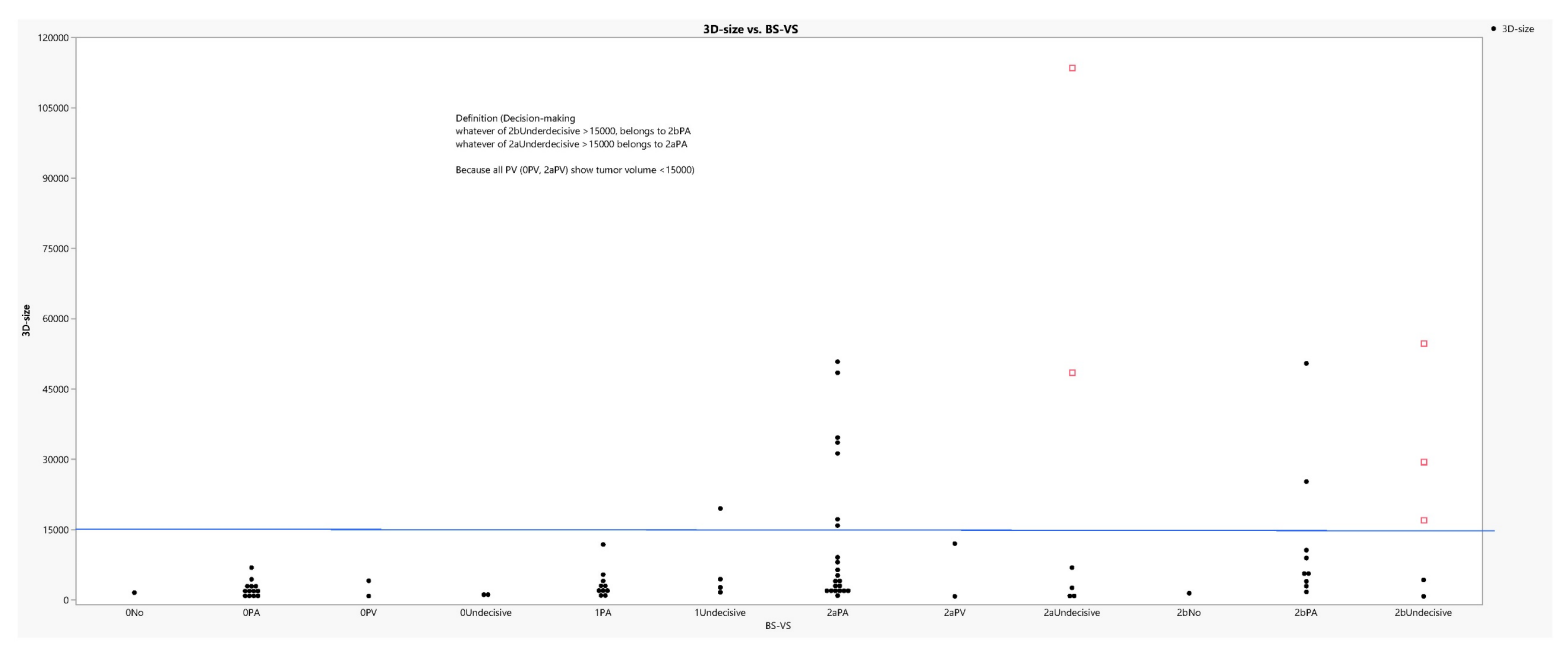
Tumor size distribution according to the concatenated categories of variable BS-VS. Values in red denote distant undecisive values and above the 15000mm^3^ critical level.

**Figure 11:**
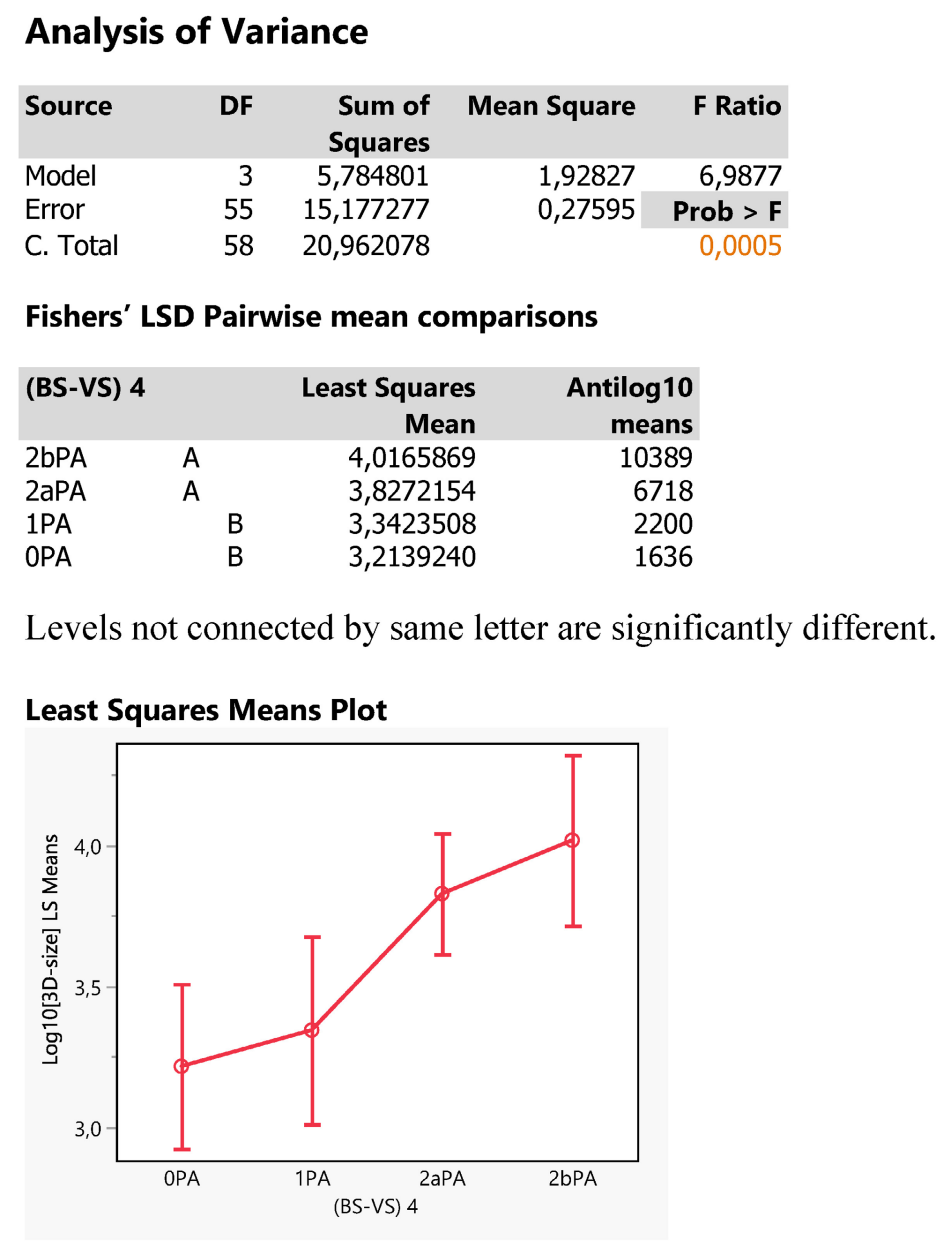
Statistical report the Response Log10[3D-size] as affected by the 4 Tokoro classifications (BS-VS 4).

**Figure 14:**
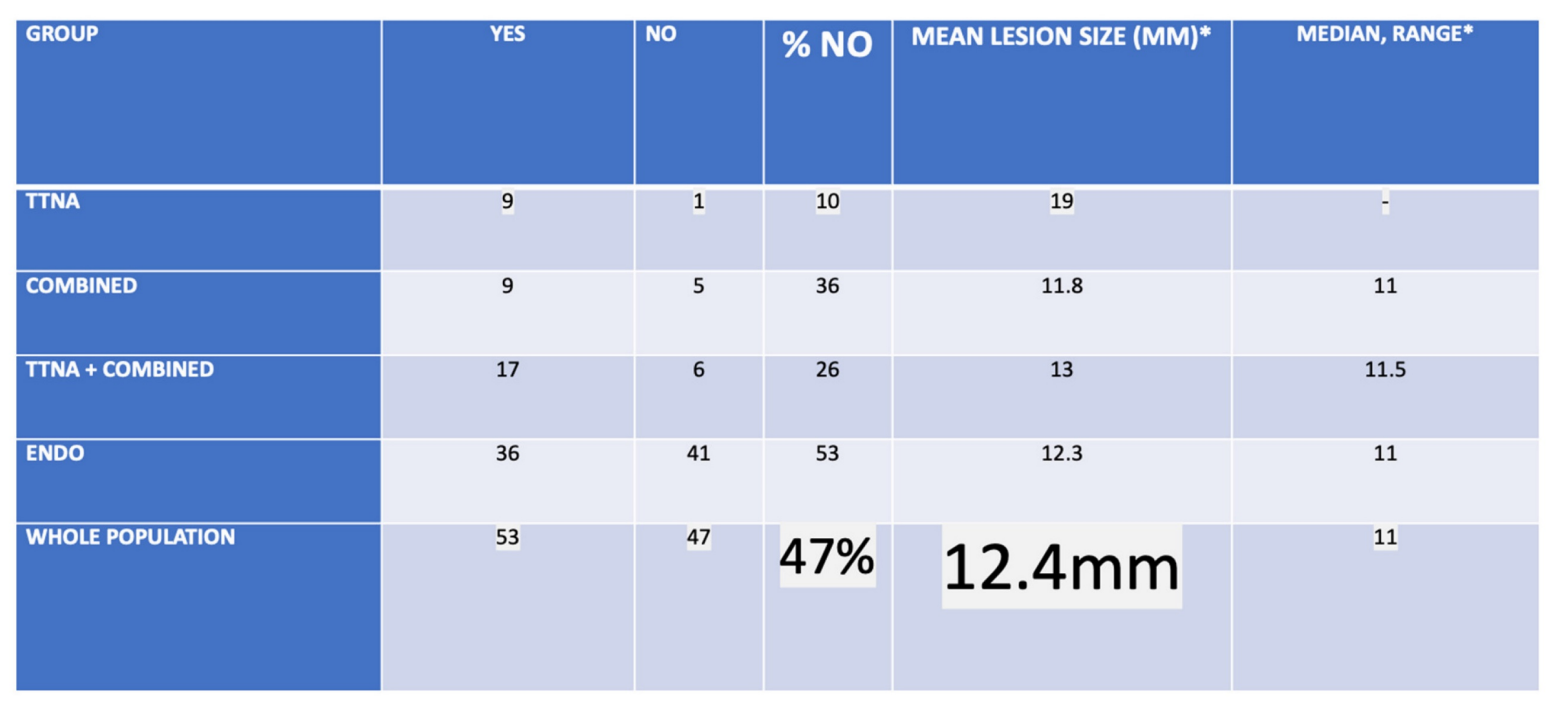
XR visibility in all subgroups.

**Figure 15:**
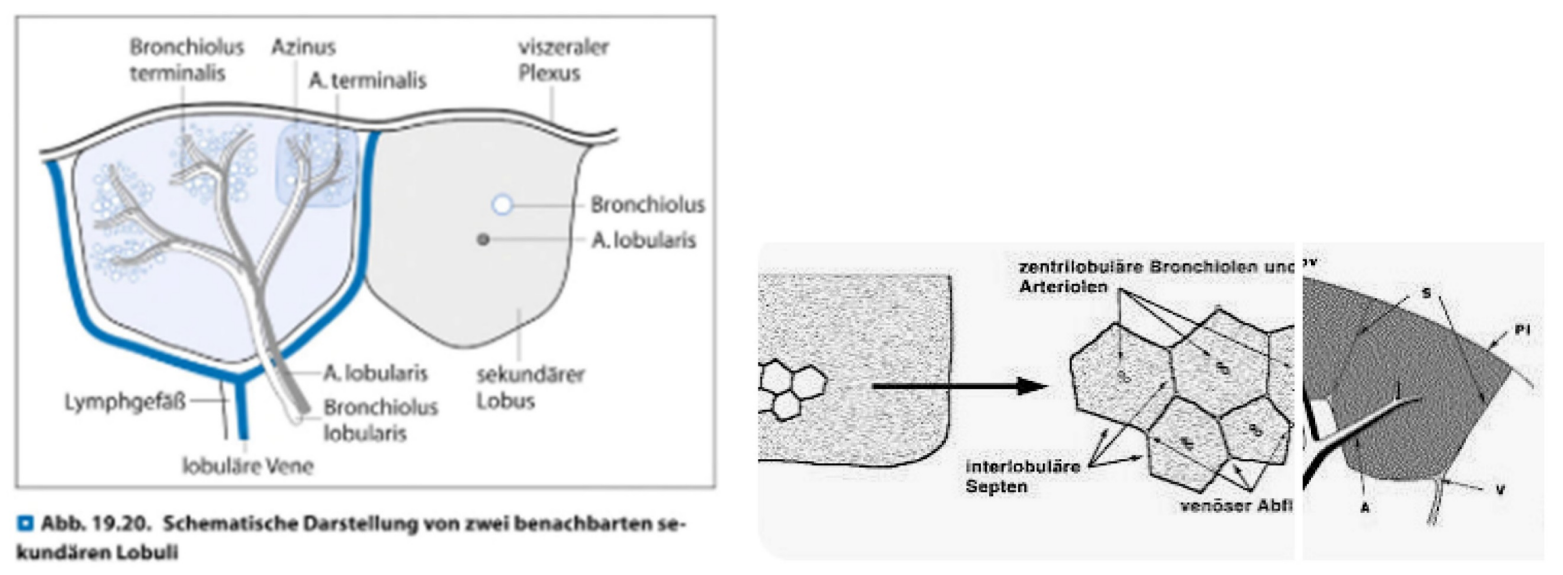
Anatomy of a secondary lobule including artery lobularis as the central branch of a secondary lobule fed by a pulmonary artery branch.

**Table 1:** Patient and clinical characteristics of pure endobiopsy group. In 67 patients 77 lesions biopsied (77% of the whole registry)

Variable (patients)	67
**Age** yrs. median (range)	68 (16-91)
**Gender**	
Male, number (%)	47 (70.1%)
Female, number (%)	20 (29.9%)
**BMI** Mean (range)	25.7 (11.9-38.6)
**Variable** (lesions) Size of the lesion (diameter)	**77**
mm, mean	17.7
mm, median	14
**Lesions grouped by size**	
>2cm (n, mean in mm)	45 (31)
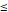 2cm (n, mean in mm)	32 (12.8)
** Location of the lesions**	
Upper lobe	36
Lower lobe	41
**CT Bronchus sign**	
0	18
1	14
2b	15
2a	30
**CT Vessel sign**	
0	2
1	4
2b	17
2a	54
**rEBUS sign**	
0	10
1	9
2b	21
2a	37
**Histology**	
Benign	47
Malignant	30
**Diagnostic Yield**	
Correct	72
False	5

**Table 2:** Diagnosis made by endobiopsy

	No. of Lesions	
**Diagnosis**	n	%
**Malignant**		
Non-small cell lung cancer		
Adenocarcinoma	9	11.7
Squamous	8	10.4
Small cell lung cancer	1	1.3
Carcinoid tumor of the lung	1	1.3
Metastatic to the lung		
RCC	1	1.3
CRC	1	1.3
Esophagus	2	2.6
Prostate	1	1.3
Nonspecified	1	1.3
**Benign**		
Pneumonia		
COP	7	9.1
NSIP	1	1.3
Lymphocytic interstitial	1	1.3
Infection		
Fungal		
Aspergillus	1	1.3
Abscess	1	1.3
Granuloma		
Inflammatory	1	1.3
Eosinophilic	1	1.3
Infarction	4	5.2
Inflammation	7	9.1
Scar		
Inflammatory	2	2.6
Elastoid	1	1.3
Necrotic	1	1.3
Nonspecified	6	7.8
Amyloidosis	1	1.3
Bronchiolitis	3	3.9
Anthracosilicotis	1	1.3
Anthracosis	10	13.0
Dysplasia	1	1.3
Abscess not specified	2	2.6

**Table 3:** Clinical characteristics by diagnostic yield of pure endobiopsy

		Diagnostic yield by endobiopsy		
		*Yes (n=72)*	*No (n=5)*	P value*
1. Size of nodule/mass, n (%)				
Mean size (mm)		17.7	16.6	0.46**
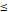 2 cm		53 (93)	4 (7)	
> 2 cm		19 (95)	1 (5)	>0.99
2. Location, n (%)				
Upper lobe		43 (96)	2 (4)	
Lower lobe		29 (91)	3 (9)	0.64
3. Lung zone, n (%)				
1		1 (100)	0	
2		25 (89)	3 (11)	
3		46 (96)	2 (4)	0.39
4. Bronchus sign, n (%)				
*Yes*		55 (93)	4 (7)	
*No*		17 (94)	1 (6)	>0.99
0		17 (94)	1 (6)	
1		13 (93)	1 (7)	
2a		27 (90)	3 (10)	
2b		15 (100)	0	0.86
5. Vessel sign, n (%)				
PA		51 (94)	3 (6)	
PV		3 (75)	1 (25)	
PA or PV		16 (94)	1 (6)	
No		2 (100)	0	0.397
6. Navigation Yield, n (%)				
Positive		63 (94)	4 (6)	
Negative		9 (90)	1 (10)	0.51

*Fisher's exact test is used, **Wilcoxon test for mean size

**Table 4:** Clinical characteristics by diagnostic yield of pure endobiopsy for peripheral lesions (zone 3)

	Diagnostic Yield				
Independent variables	Correct (n=46)	False (n=2)	Odds ratio	95% CI	*P* value*
Vessel sign, n (%)					
*Positive*	45 (96)	2 (4)			
*Negative*	1 (100)	0 (0)	0	0 - 884.6	>0.99
Bronchus sign					
*Positive*	33 (94)	2 (6)			
*Negative*	13 (100)	0 (0)	0	0 - 114.58	>0.99

*Fisher's exact test is used

**Table 5:** Clinical characteristics by navigational yield of endobiopsy

		Navigation Yield		
		*Yes (n=67)*	*No (n=10)*	P value*
**Size of nodule/mass, n (%)**				
Mean size		18.4	13.2	0.0779
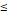 2 cm		48 (84)	9 (6)	
> 2 cm		19 (95)	1 (5)	0.4391
**Lung zone, n (%)**				
1		1 (100)	0	
2		27 (96)	1 (4)	
3		39 (81)	9 (19)	0.20
**Bronchus sign, n (%)**				
Class 0		12 (67)	6 (33)	
Class 1		11 (79)	3 (21)	
Class 2b		14 (93)	1 (7)	
Class 2a		30 (100)	0	**0.0018**
**Vessel sign, n (%)**				
Class 0 (No vessel)		1 (50)	1 (50)	
Class 1 (PV)		3 (75)	1 (12)	
Class 2b (undecisive)		15 (88)	2 (12)	
Class 2a (PA)		48(89)	6 (11)	0.27

*Fisher's exact test is used

**Table 6:** Clinical characteristics by navigational yield of endobiopsy for peripheral lesions (zone 3)

	Navigation Yield				
Independent variables	Correct (n=39)	False (n=9)	Odds ratio	95% CI	*P* value*
Vessel sign, n (%)					
*Positive*	39 (83)	8 (17)			
* Negative*	0 (0)	1 (100)	inf	0.11 - inf	0.188
Bronchus sign, n (%)					
*Positive*	31 (89)	4 (11)			
* Negative*	8 (62)	5 (38)	**4.65**	0.80 - 29.65	**0.048**

*Fisher's exact test was used

**Table 7:** Multivariate logistic regression model for diagnostic yield of endobiopsy group

Independent variables	Odds ratio	95% CI	*P* value*
Vessel sign (0 is ref)	7.33	0.27 - 196.9	0.172
Vessel sign PA (0 is ref)	1.68	0.39 - 6.58	0.457
Bronchus sign (0 is ref)	6.88	1.71 - 30.7	0.007
Vessel sign classes			
* PA*	(ref)	(ref)	(ref)
* PV*	0.38	0.04 - 8.25	0.426
* Undecisive*	0.94	0.19 - 6.86	0.941
* No*	0.13	0.005 - 3.43	0.160
* PV*	(ref)	(ref)	(ref)
* Undecisive*	2.50	0.10 - 36.54	0.506
* No*	0.33	0.005 - 14.24	0.547
* Undecisive*	(ref)	(ref)	(ref)
* No*	0.13	0.004 - 4.25	0.209
			
Bronchus sign classes			
*0*	(ref)	(ref)	(ref)
* 1*	1.83	3.82e-01 - 10.41	0.460
* 2a*	1.57e+08	4.39e-60 - NA	0.992
* 2b*	7.00	1.00 - 142.25	0.091
1	(ref)	(ref)	(ref)
* 2a*	8.57e+07	2.39e-60 - NA	0.993
* 2b*	3.82	4.21e-01 - 83.32	0.273
2a	(ref)	(ref)	(ref)
* 2b*	4.45e-08	NA - 1.60e+60	0.993
*Class 2a and 2b together*			
*0*	(ref)	(ref)	(ref)
* 1*	1.83	0.38 - 10.41	0.460
* 2*	22.00	3.33 - 437.33	0.006
1	(ref)	(ref)	(ref)
* 2*	12.00	1.39 - 256.10	0.039

*Logistic regression analysis was used

**Table 8:** Logistic regression model for navigational yield of endobiopsy group in lung zone 3

	Navigation Yield in lung zone 3				
Independent variables	Correct (n=39)	False (n=9)	Odds ratio	95% CI	*P* value*
Vessel sign, n (%)					
*Positive*	39 (83)	8 (17)			
* Negative (ref)*	0 (0)	1 (100)	7.63e+07	4.03e-205 - NA	0.994
Vessel sign PA, n (%)					
*Positive*	30 (83)	6 (17)			
* Negative (ref)*	9 (75)	3 (25)	1.67	0.31 - 7.79	0.525
Bronchus sign, n (%)					
*Positive*	31 (89)	4 (11)			
* Negative (ref)*	8 (62)	5 (38)	4.84	1.06 - 23.98	0.043

*Logistic regression analysis was used

**Table 9:** Logistic regression model for diagnostic yield of endobiopsy group

Independent variables	Odds ratio	95% CI	*P* value*
Vessel sign (0 is ref)	3.29e-07	NA - 3.73e+183	0.996
Vessel sign PA (0 is ref)	1.62	0.20 - 10.46	0.612
Bronchus sign (0 is ref)	0.81	0.04 - 5.94	0.854
Vessel sign classes			
* PA*	(ref)	(ref)	(ref)
* PV*	1.77e-01	1.55e-02 - 4.15	0.182
* Undecisive*	9.41e-01	1.11e-01 - 19.76	0.959
* No*	2.50e+06	1.75e-183 - NA	0.996
* PV*	(ref)	(ref)	(ref)
* Undecisive*	5.33e+00	1.78e-01 - 164.46	0.279
* No*	1.42e+07	1.16e-125 - NA	0.995
* Undecisive*	(ref)	(ref)	(ref)
* No*	2.66e+06	2.17e-126 - NA	0.996
***Class 0VS extracted because of 0 values***			
* PA*	(ref)	(ref)	(ref)
* PV*	0.18	0.02 - 4.15	0.182
* Undecisive*	0.94	0.11 - 19.76	0.959
* PV*	(ref)	(ref)	(ref)
* Undecisive*	5.33	0.18 - 164.46	0.279
**Bronchus sign classes**			
*0*	(ref)	(ref)	(ref)
* 1*	7.65e-01	2.84e-02 - 20.57	0.854
* 2a*	5.29e-01	2.51e-02 - 4.52	0.595
* 2b*	1.85e+07	2.04e-124 - NA	0.995
1	(ref)	(ref)	(ref)
* 2a*	6.92e-01	3.25e-02 - 6.02	0.760
* 2b*	2.42e+07	2.67e-124 - NA	0.995
2a	(ref)	(ref)	(ref)
* 2b*	3.49e+07	3.86e-124 - NA	0.995
***Class 2a and 2b together because of 0 values***			
*0*	(ref)	(ref)	(ref)
* 1*	0.77	0.03 - 20.57	0.854
* 2*	0.82	0.04 - 6.96	0.870
1	(ref)	(ref)	(ref)
* 2*	1.08	0.05 - 9.26	0.951

*Logistic regression analysis was used

**Table 10:** Logistic regression model for diagnostic yield of endobiopsy group in lung zone 3

Diagnostic Yield in lung zone 3					
Independent variables	Correct (n=46)	False (n=2)	Odds ratio	95% CI	*P* value*
Vessel sign, n (%)					
*Positive*	45 (96)	2 (4)			
* Negative (ref)*	1 (100)	0 (0)	5.29e-07	NA - inf	0.997
Vessel sign PA, n (%)					
*Positive*	35 (97)	1 (3)			
* Negative (ref)*	11 (92)	1 (8)	3.18	0.12 - 85.09	0.427
Bronchus sign, n (%)					
*Positive*	33 (94)	2 (6)			
* Negative (ref)*	13 (100)	0 (0)	5.25e-08	0.12 - 85.09	0.996

*Logistic regression analysis was used

**Table 11:** Multinominal logistic regression model for diagnostic yield of endobiopsy group

Independent variables	Odds ratio	95% CI	*P* value*
Vessel Sign vs Bronchus sign			
* Vessel sign (0 is ref)*	4.11e-03	4.78e-39 - 3.54e+33	0.896
* Bronchus sign (0 is ref)*	8.44e-01	8.80e-02 - 8.09	0.883
Vessel Sign PA vs Bronchus sign			
* Vessel sign PA (0 is ref)*	**1.61**	0.25 - 10.37	0.615
* Bronchus sign (0 is ref)*	0.82	0.09 - 7.87	0.863
Vessel Sign classes vs BS			
* VS-No (ref)*			
* VS-PA*	6.49e-03	1.77e-35 - 2.39e+30	0.895
* VS-PV*	1.01e-03	2.69e-36 - 3.81e+29	0.857
* VS-Undecisive*	6.42e-03	1.71e-35 - 2.41e+30	0.895
* Bronchus sign (0 is ref)*	6.28e-01	5.59e-02 - 7.05	0.706
Vessel Sign vs BS classes			
* BS-0 (ref)*	8.12e-01	4.62e-02 - 1.43e+01	0.887
* BS-1*	5.62e-01	5.38e-02 - 5.87	0.631
* BS-2a*	5.35e+03	1.47e-63 - 1.94e+70	0.913
* BS-2b*	5.29e-03	7.14e-50 - 3.91e+44	0.924
* Vessel sign (0 is ref)*			

*Multinominal LR model was used

**Table 12:** Multinominal logistic regression model for navigational yield of endobiopsy group

Independent variables	Odds ratio	95% CI	*P* value*
Vessel Sign vs Bronchus sign			
* Vessel sign (0 is ref)*	5.69	0.24 - 135.64	0.282
* Bronchus (0 is ref)*	**6.58**	0.04 - 0.64	**0.010**
Vessel sign PA vs BS			
* Vessel sign PA (0 is ref)*	1.93	0.44 - 8.48	0.383
* Bronchus sign (0 is ref)*	**7.20**	1.72 - 30.15	**0.007**

*Multinominal LR model was used

**Table 13:** Tally of the parameters under study.

			Concatenation		
**NY (rEBUS)**	N		**BS-VS**	N	
0	10		0No	1	
1	67		0PA	13	
**Bronchus Sign (BS)**			0PV	2	
0	18		0Undecisive	2	
1	14		1PA	10	
2a	30		1Undecisive	4	
2b	15		2aPA	24	22+2
**Vessel Sign (VS)**			2aPV	2	
No	2		2aUndecisive	4	`**6**-2
PA	59		2bNo	1	
PV	4		2bPA	12	9+3
Undecisive	12		2bUndecisive	2	`**5**-3
**Lung zone**				77	
1	1				
2	28				
3	48				

**Table 14:** Simple and cross-tabulated arrays of the most important variables of the study.

(BS-VS) 4			BSVS				NY (rEBUS)				
0PA	13		0PA	13			0		1		
1PA	10		BS^	46			**Lung zone**				
2aPA	24					**BSVS**	2	3	2	3	
2bPA	12					0PA	0	**4**	2	**7**	13
						BS^	0	2	19	25	46
											59
							BS^=BS*PA(1,2a,2b)				

**Table a:** Patient characteristics 1

Age	BMI	Mean (mm)	3D-size	NY (rEBUS)	Lung zone	BSVS
58	28,7	11	1287	1	3	0PA
59	27,8	13	1950	0	3	0PA
78	27,7	11	1430	1	3	0PA
84	20,8	12	1872	1	3	0PA
68	30,1	19	6840	1	3	0PA
75	20,7	16	4352	1	3	0PA
69	29,2	14	2880	1	3	0PA
91	31,3	13	2184	1	3	0PA

**Table b:** Patient characteristics 2

Age	BMI	Mean (mm)	3D-size	NY (rEBUS)	Lung zone	BSVS
73	25,8	11	1155	0	3	0PA
81	24,8	10	891	0	3	0PA
		10	864	1	3	
		9	640	0	3	0PA
63	22,6	10	714	1	3	

**Table c:** Patient characteristics 3

Age	BMI	Mean (mm)	3D-size	NY (rEBUS)	Lung zone	BSVS
70	24,2	9	768	0	2	
62	22,9	10	900	1	2	0PA
78	22,3	9	720	1	2	
52	20,8	9	720	1	2	
69	29,8	9	810	1	2	0PA

**Table 15:** Patient and clinical characteristics of whole study population

Variable (patients)	90
Age yrs. median (range)	69 (16-91)
Gender	
Male, number (%)	61 (68)
Female, number (%)	29 (32)
BMI Mean (range)	25.2 (11.9-38.6)
Variable (lesions)	100
Size of the lesion (diameter) mm, mean (range)	19.7
median	16
Location of the lesions	
Upper lobe	62
Lower lobe	38
Histology	
Benign	53
Malignant	47
Diagnostic Yield	
Correct	95
False	5

**Table 16:** Diagnostic performance. *Spec = TN/(FP+TN). PPV = TP/(TP+FP). NPV = TN/(TN+FN). Accuracy = (TP+TN)/(TN+FP+FN+TP)*

Group	Malignant (n)		TP	TN	FP	FN
TTNA + Combined	5+12=17		17	6	0	0
Endo	30		25	47	0	5
WHOLE POPulation	47		42	53	0	5
Group	Spec	PPV	NPV	ACCURACY		
TTNA + Combined	1	1	1	1		
Endo	1	1	0.90	0.94		
WHOle population	1	1	0.91	0.95		

**Table 17:** Analysis of size differences. Sizes of lesions (n=100).

median, mean size endobiopsy group (mm):	14, 17.7
median, mean size TTNA group (mm):	27, 31
median, mean size Combined group (mm):	18.5, 23
median, mean size of TTNA+Combined (mm):	25, 26.4
**median, mean size of whole group (mm):**	**16, 19.7**
**Size comparison between endobiopsy group and TTNA+Combined groups**	
Wilcoxon rank sum test with continuity correction: W=1254.5; p=0.002493	


**Table 18:** Statistical analysis of unfavorable bronchus or vessel sign patterns

a) BS- or VS- or VS-PV				
Independent variables	(n)	Odds ratio	95% CI	*P* value*
Negative bronchus sign (positive is ref) Com+TTNA (Endo is ref)	22	0.73	0.19 - 2.26	0.606
Negative vessel sign (positive is ref) Com+TTNA (Endo is ref)	8	14.06	2.94 - 102.23	**0.002**
Positive VS-PV sign (negative is ref) Com+TTNA (Endo is ref)	5	0.87	0.04 - 6.28	0.902
*logistic regression model				
b) Multivariate				
Independent variables		Odds ratio	95% CI	*P* value*
Com+TTNA (Endo is ref)				
BS-		0.63	0.16 - 2.43	0.497
VS-		14.86	2.68 -82.35	**0.002**
VS-PV		1.42	0.14 - 14.60	0.767
*multinominal logistic regression model				

**Table 19:** Percentage of class 2 Bronchus sign

Group	Class	Class/Total	Percentage
Combined	BS 2a+2b	6/14	43%
TTNA	BS 2a+2b	2/8	25%
TTNA + Combined	BS 2a+2b	8/22	36%
Endo	BS 2a+2b	45/77	58%

**Table 20:** Analysis of XR visibility

Group	YEs	NO	% NO	MEAN LESIOn SIZE (MM)*	MEDIAN, range*
TTNA	8	1	19	19	-
Combined	9	5	35	11.8	11 (8-18)
TTNA + Combined	17	6	26	13	11.5 (8-19)
Endo	36	41	53	12.3	11 (9-32)
WHOLE POPulation	53	47	47	12.4	11 (8-32)
*Of NO group					
Group	MEDIAN YEs		MEdian NO		p-value*
TTNA + Combined	27		11.5		0.001816
Endo	20.5		11		7.066e-10
WHOLE POPulation	23		11		3.613e-13
*Wilxocon rank sum test (Mann-Whitney U test) is used					

## Abbreviations

AF: Augmented fluoroscopy
BMI: Body mass index
BPT: Bronchopulmonary trias
BS: Bronchus sign
C-ARM: Normal fluoroscopy with the help of a c-arm
CBCT: Conebeam Computertomography
CBCT TIL: CBCT for tool-in-lesion control
CI: Confidence interval
CRC: Colorectal cancer
CT: Computertomography
DY: Diagnostical yield
EWC: Extended working channel
FN: False negative
FNA or FNB: Fine Needle Aspiration or Biopsy
FU: Follow Up
HRCT: High resolution Computertomography
IP: Interventional Pulmonology
iSPN: Incidental solitary pulmonary nodule
LL: Lower lobe
NY: Navigational yield (by any visualization of rEBUS)
Centred position: Tool is in the inner 2/3 of the rEBUS path AND in the inner 2/3 of the AF volume of an iSPN (which comes near to the very often used term concentric)
Inside position: Tool is in the outer 2/3 of the rEBUS path AND in the outer 2/3 of the AF volume of an iSPN (which comes near - but in some cases still unequal - to the very often used term excentric)
Tangential position: rEBUS can visualize the iSPN by touching the outer rim or in the very nearest surrounding of the iSPN resulting in a typical 'black shadow' picture
OR: Odds ratio
PA: Pulmonary artery
PV: Pulmonary vein
paCO_2_: pulmonary arterial carbon dioxide
rEBUS: Radial endobronchial ultrasound classes for navigational yield (NY) by rEBUS and AF: 0: no/negative NY; 1: rEBUS adjacent to iSPN (tangential view or black shadow picture); 2: rEBUS leading into an iSPN; 2a: rEBUS leading into a centred intratumoral position of an iSPN (inner 2/3 of the volume defined by rEBUS position and AF); 2b: rEBUS leading into the outer 1/3 of the volume of an iSPN defined by rEBUS position and AF (inside position but not centred)
RCC: Renal cell carcinoma
RUFB: Repeated use fibre bronchoscope
SD: Standard deviation
SL: Secondary lobulus
TP: True positive
TTNA: Transthoracic needle approach Tsokoro classification (extended) for CT/CBCT classes of bronchus sign (BS): 0: no/negative BS; 1: BS adjacent to iSPN; 2: BS leading into an iSPN; 2a: BS leading into a centred intratumoral position of an iSPN (inner 2/3 of the volume); 2b: BS leading into the outer 1/3 of the volume of an iSPN hereby leading at least inside
UTN: Ultrathin
UL: Upper lobe
VS: Vessel sign classes to be classified on CT/CBCT: 0: No branch of any vessel leading into or touching an iSPN; 1: A branch of a pulmonary vein as vessel sign leading into or touching an iSPN; 2a: A branch of a pulmonary artery as vessel sign leading into or touching an iSPN; 2b: A vessel branch of unclear origin leading into or touching an iSPN. Possible 16 combinations to describe a nodule in the setting of bronchus and vessel sign classes (Class, Mode): 0, BS: 0BS means no bronchus sign; 1, BS: 1BS means a bronchus sign adjacent to the iSPN; 2b, BS: 2bBS means a bronchus sign ends in the outer 1/3 of the iSPN volume; 2a, BS: 2aBS means a bronchus sign ends in the inner 2/3 of the iSPN volume combined with: 0 VS: 0VS means no vessel sign visible towards the iSPN; 1 VS: 1VS means a PV branch leads into or touching the iSPN (PV); 2b into or touching the iSPN (ANY); 2a VS: 2aVS means a PA branch leads into tor touches the iSPN (PA)
XR: Fluoroscopy
