# Supplementary material for: Vessel sign analysis paves the way to optimized CBCT application in interventional pulmonology: COMBINED algorithm as a one-stop-shop
Source: J Cancer. 2025 Mar 21;16(7):2124–44. doi: 10.7150/jca.109996 (PMC12036083; doi:10.7150/jca.109996)
Supplement: Supplementary file 1 — Supplementary information. [file jcav16p2124s1.pdf]

Prediction Profiler

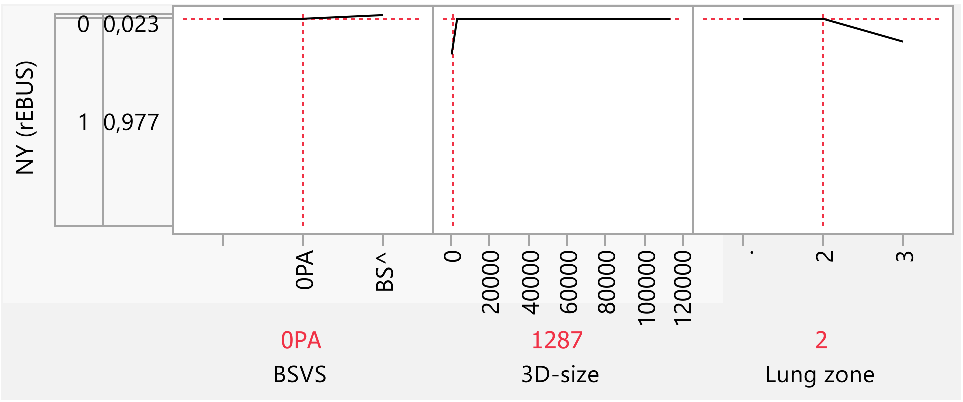

Remembered Settings

Setting	BSVS	3D-size	Lung zone	NY (rEBUS)
Setting 1	0PA	1287	2	0,9769
Setting 2	0PA	1287	3	0,8701
Setting 3	0PA	1078	2	0,9769
Setting 4	0PA	1078	3	0,4732

In terms of clinical characteristics in the diagnostic yield of endobioscopy the findings were sorted according to a confusion matrix and a ROC curve to the following results:

The **Confusion Matrix** is a two-way classification of actual and predicted responses and the present partition analysis produces the 2x2 contingency table by numbers and rates.

Metrics

		Predicted		Rates	
**Method**	**Actual**	**0**	**1**	**0**	**1**
Partition	1	2	65	0,03	0,97
Partition	0	3	7	0,30	0,70

Method	TP	FN	FP	TN	Sensitivity	Specificity	Precision	Accuracy	F1	MCC
Partition	65	2	7	3	0,9701	0,3000	0,9028	0,8831	0,9353	0,3685

Where 1 is considered Positive. Probability Threshold: 0,500

Definitions

TP = True Positives
FN = False Negatives
TN = True Negatives
FP = False Positives
Sensitivity = TP/(TP+FN)
Specificity = TN/(TN+FP)
Precision = TP/(TP+FP)
Accuracy = (TP+TN)/(TP+TN+FP+FN)
F1 = 2*TP/(2TP+FP+FN)
MCC = (TP*TN-FP*FN)/sqrt((TP+FP)*(TP+FN)*(TN+FP)*(TN+FN))

The Receiver Operating Characteristic (ROC) curve displays the efficiency of the model's fitted probabilities to sort the response levels of NY. Judging from the track of the two lines, the distance from the

diagonal line (equal to 50% rejection) and mostly from the high AUC value (0,895), it follows that the model performs very adequately and could be safely used for new entries of patient records to predict, in high probability, correctly the NY values.

Receiver Operating Characteristic on Data

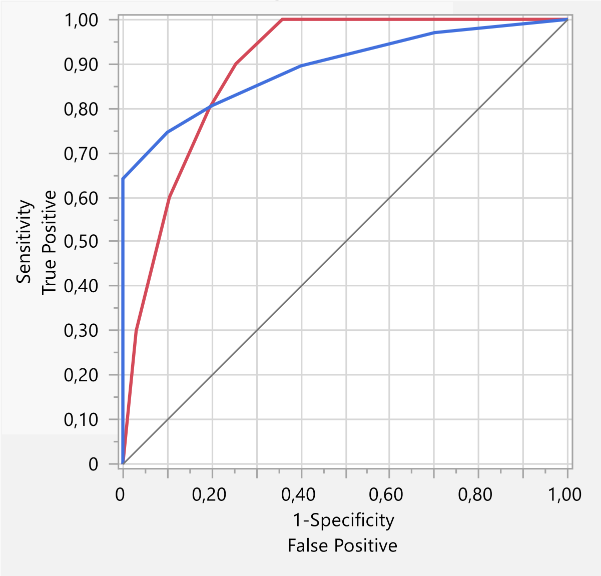

NY (rEBUS)	Area
0	0,8925
1	0,8925

It is noteworthy, that the decision tree of the study achieves to detect the 13 0PA *(0BS*2aVS)* counts in three discrete nodes with specific attributes each.

Exhibit 3. Statistical report of data partitioning in nodes using NY as a response variable and tumor 3D-size, lung zone and BSVS (0PA, BS^2) as predictors.

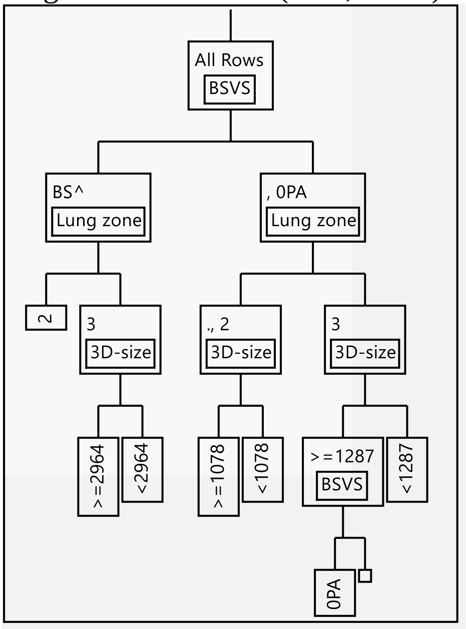

RSquare	N	Number of Splits
0,358	77	7

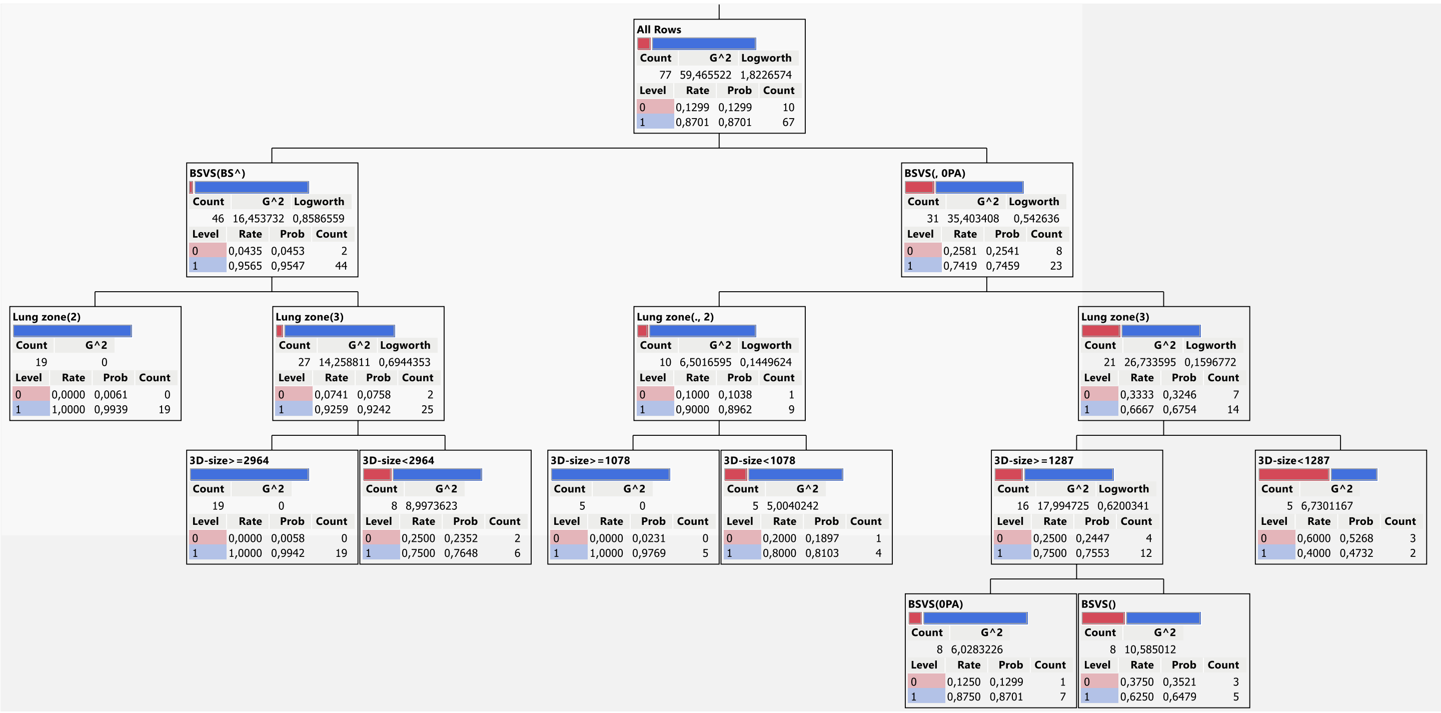

Split History

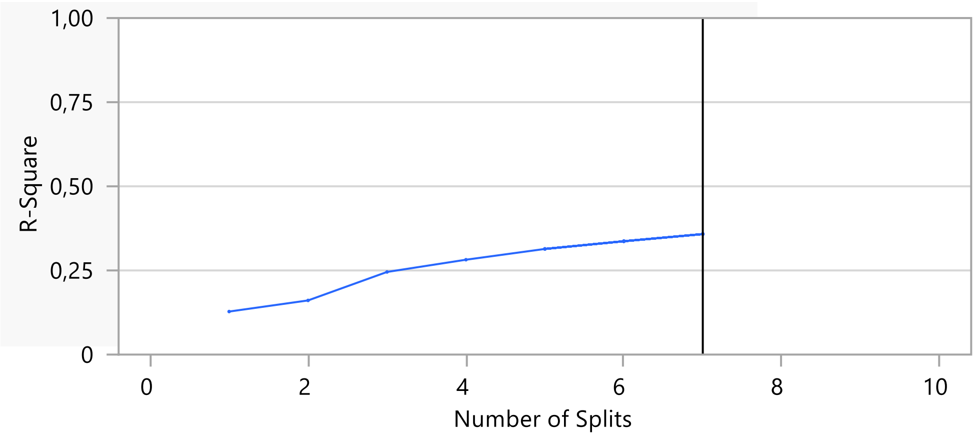

Column Contributions

Term	Number of Splits	G^2		Portion
BSVS	2	8,9897723		0,4064
3D-size	3	8,76783749		0,3964
Lung zone	2	4,36307471		0,1972

Leaf Report

Response Probabilities

Leaf Label	0		1	
BSVS(BS^)&Lung zone(2)	0,0061		0,9939	
BSVS(BS^)&Lung zone(3)&3D-size>=2964	0,0058		0,9942	
BSVS(BS^)&Lung zone(3)&3D-size<2964	0,2352		0,7648	
BSVS(, 0PA)&Lung zone(., 2)&3D-size>=1078	0,0231		0,9769	
BSVS(, 0PA)&Lung zone(., 2)&3D-size<1078	0,1897		0,8103	
^&Lung zone(3)&3D-size>=1287&BSVS(0PA)	0,1299		0,8701	
^&Lung zone(3)&3D-size>=1287&BSVS()	0,3521		0,6479	
BSVS(, 0PA)&Lung zone(3)&3D-size<1287	0,5268		0,4732	

Response Counts

Leaf Label	0	1
BSVS(BS^)&Lung zone(2)	0	19
BSVS(BS^)&Lung zone(3)&3D-size>=2964	0	19
BSVS(BS^)&Lung zone(3)&3D-size<2964	2	6
BSVS(, 0PA)&Lung zone(., 2)&3D-size>=1078	0	5
BSVS(, 0PA)&Lung zone(., 2)&3D-size<1078	1	4
^&Lung zone(3)&3D-size>=1287&BSVS(0PA)	1	7
^&Lung zone(3)&3D-size>=1287&BSVS()	3	5
BSVS(, 0PA)&Lung zone(3)&3D-size<1287	3	2
